# Identification, Validation and Utilization of Novel Nematode-Responsive Root-Specific Promoters in Arabidopsis for Inducing Host-Delivered RNAi Mediated Root-Knot Nematode Resistance

**DOI:** 10.3389/fpls.2017.02049

**Published:** 2017-12-12

**Authors:** Atul Kakrana, Anil Kumar, Viswanathan Satheesh, M. Z. Abdin, Kuppuswamy Subramaniam, R. C. Bhattacharya, Ramamurthy Srinivasan, Anil Sirohi, Pradeep K. Jain

**Affiliations:** ^1^ICAR-National Research Centre on Plant Biotechnology, New Delhi, India; ^2^Center for Bioinformatics and Computational Biology, University of Delaware, Newark, DE, United States; ^3^Department of Biotechnology, Faculty of Science, Centre for Transgenic Plant Development, Jamia Hamdard University, New Delhi, India; ^4^Department of Biotechnology, Indian Institute of Technology Madras, Chennai, India; ^5^Division of Nematology, ICAR-Indian Agricultural Research Institute, New Delhi, India

**Keywords:** *Arabidopsis*, HD-RNAi, *in silico* analysis, nematode-responsive genes, promoter analysis, root-specific genes

## Abstract

The root-knot nematode (RKN), *Meloidogyne incognita*, is an obligate, sedentary endoparasite that infects a large number of crops and severely affects productivity. The commonly used nematode control strategies have their own limitations. Of late, RNA interference (RNAi) has become a popular approach for the development of nematode resistance in plants. Transgenic crops capable of expressing dsRNAs, specifically in roots for disrupting the parasitic process, offer an effective and efficient means of producing resistant crops. We identified nematode-responsive and root-specific (NRRS) promoters by using microarray data from the public domain and known conserved *cis*-elements. A set of 51 NRRS genes was identified which was narrowed down further on the basis of presence of *cis*-elements combined with minimal expression in the absence of nematode infection. The comparative analysis of promoters from the enriched NRRS set, along with earlier reported nematode-responsive genes, led to the identification of specific *cis*-elements. The promoters of two candidate genes were used to generate transgenic plants harboring promoter GUS constructs and tested *in planta* against nematodes. Both promoters showed preferential expression upon nematode infection, exclusively in the root in one and galls in the other. One of these NRRS promoters was used to drive the expression of *splicing factor*, a nematode-specific gene, for generating host-delivered RNAi-mediated nematode-resistant plants. Transgenic lines expressing dsRNA of *splicing factor* under the NRRS promoter exhibited upto a 32% reduction in number of galls compared to control plants.

## Introduction

*Meloidogyne incognita*, the southern root-knot nematode (RKN), is an obligatory sedentary parasite that infects thousands of plant species. The life cycle of *M. incognita* (Kofoid and White, 1919) starts with the hatching of egg in the soil, maturing into pre-parasitic second-stage juvenile, which later penetrates the root tip, migrates along the vascular cylinder of the plant root, becomes sedentary and forms a feeding site. RKN infestation causes an estimated annual crop loss of hundreds of billions of dollars (Abad et al., 2008; Elling, 2013). The demonstration of host-delivered RNA interference (HD-RNAi) in plant-parasitic nematodes (PPNs) offers an effective strategy to control nematode infection in crop plants (Tamilarasan and Rajam, 2013; Dutta et al., 2015; Dong et al., 2016; Niu et al., 2016; Banerjee et al., 2017; Kumar et al., 2017). Two house-keeping genes (*splicing factor* and *integrase*) were successfully silenced in nematodes through host-mediated RNAi in tobacco plants (Yadav et al., 2006). *Arabidopsis thaliana* (L.) *Heynth*. transgenic line expressing dsRNA of the *16D10* gene of RKN show significant reduction in the number of galls (65–90%) compared to the control (Huang et al., 2006). HD-RNAi of the putative effector gene *Mc16D10L* in potato (*Solanum tuberosum* L.) and *Arabidopsis* show significant resistance to *M. chitwoodi* (Dinh et al., 2014).

RNAi based approaches for nematode control largely use the constitutive promoter CaMV35S for dsRNA production in host plants (Tamilarasan and Rajam, 2013). However, the utility of CaMV35S “constitutive” promoter in driving expression of RNAi constructs is highly debated due to possibility of off-targets effects (Goddijn et al., 1993; Urwin et al., 1997; Bertioli et al., 1999). Moreover, transgenic plants with a strong constitutive expression of stress-responsive genes often suffer from undesirable phenotypes. The stress-tolerant transgenic *Arabidopsis* expressing *35S::DREB1A* displayed growth retardation and severe reduction in seed production (Liu et al., 1998; Yamaguchi-Shinozaki and Shinozaki, 2001). Similar observations were made in transgenic tomato (*Solanum lycopersicum* L.), rice (*Oryza sativa* L.), and tobacco (*Nicotiana tabacum* L.) plants.

Since expression of transgenes driven by constitutive promoters, especially for a HD-RNAi approach is less desired due its potential off-targets effects, it is imperative that stress-inducible and tissue-specific promoters are identified to minimize these effects. Several researchers have stressed the need for identification of tissue-specific nematode-responsive promoters (Rosso et al., 2009). Such conditional promoters responsive to *M. incognita* with preferential expression only in target tissues like roots are likely to be more effective in developing RNAi-based resistance. This strategy, therefore, can also reduce the cost for *in vivo* dsRNA expression (Bakhetia et al., 2005). Studies on nematode-responsive and root-specific (NRRS) promoters are very limited and largely based on promoter tagging and mutant line analysis. The first NRRS expression was reported in *TobRB7*, a tonoplast intrinsic protein (*TIP*) gene from tobacco that was selectively active in infested root tissue cells and was induced during the feeding cell development (Opperman et al., 1994). Similar to *TOBRB7*, a strawberry gene *FARB7* showed near root-specific expression. Though, *FARB7* shared regulatory elements with *TOBRB7* (Vaughan et al., 2006), its response to nematode still needs to be validated. Also, three promoters of *Arabidopsis*, TUB-1, ARSK1, and RPL16A have been identified, which drive expression of cystatin mainly to the roots and eventually deliver significant level of resistance against *M. incognita* (Lilley et al., 2004).

To investigate the differential expression pattern of genes in response to nematodes, a number of microarray analyses have been performed in the last decade (Portillo et al., 2013). In a few such experiments, the whole root was considered (Hammes et al., 2005; Alkharouf et al., 2006; Ithal et al., 2007; Klink et al., 2007) and some analysis focused on nematode feeding sites enriched samples only (Xiao and Xue, 2001; Bar-Or et al., 2005; Jammes et al., 2005; Fuller et al., 2007; Schaff et al., 2007; Barcala et al., 2010). Recently, NEMATIC (NEMatode-*Arabidopsis* Transcriptomic Interaction Compendium) tool was launched, which uses transcriptome data for studying the interaction between *Arabidopsis* and plant-endoparasitic nematodes (Cabrera et al., 2014). Furthermore, in spite of several studies that examined differential promoter activity in nematode feeding cells (Opperman et al., 1994; Escobar et al., 1999), no specific regulatory elements responsible for low basal expression have been identified. Putative *cis*-elements present in nematode-responsive promoters such as ERE, Wun-Motif, EIRE (Sukno et al., 2006) and P-Box (Escobar et al., 1999) were identified by the comparative analysis with regulatory elements databases but their role during nematode interaction is yet to be confirmed.

The current work describes a computational approach to identify nematode-responsive root-specific (NRRS) promoters and demonstrates their utility in host-mediated resistance in *Arabidopsis* against *M. incognita* infection.

## Materials and methods

### Propagation of nematode

The southern root-knot nematode (RKN; *M. incognita*) culture was maintained on tomato and eggplant (*Solanum melongena L*.). Tomato and eggplant seeds were sterilized (soaked for 20 min in sterile distilled water, 5 min in 70% ethanol, and 15 min in 5% NaOCl and 0.1% Tween 20, and washed four times in sterile distilled water) and germinated on MS agar medium. After 3 weeks, the plant roots were infected with 500 second-stage juveniles (J2s) of RKN. Six weeks later, egg masses were hand-picked and hatched at 28°C in 10–15 ml of sterile water.

### Plant growth conditions and nematode infection

*Arabidopsis thaliana* (*Col*-0) seeds were surface sterilized (by immersing for 2 min in 70% alcohol and 7 min in 0.1% Mercuric chloride and 0.1% SDS) and stratified for 72 h at 4°C before germination on Gamborg's B-5 medium. Plates were covered with parafilm M® and maintained at 21°C under a 16 h light/8 h dark photoperiod. Fourteen-day-old *Arabidopsis* seedlings were transferred from the Petri plates to trays containing a sand/vermicompost/cocopeat mixture (1:1:1 w/w). After 3 weeks, each plant was inoculated with 1,000 freshly hatched J2s of RKN using a 1 ml pipette. The trays were maintained in a growth chamber at 21°C and the *M. incognita*-infected galls and complete roots of *A. thaliana* were harvested at two different intervals viz., 10 and 21 days post inoculation (dpi). The uninfected root tissue, dissected from the plants at the same time points, served as control samples. At each time point, samples were harvested from both infected and uninfected plants and frozen in liquid nitrogen and stored at −80°C until further use.

### Microarray data mining and statistical analysis

Publicly available microarray data for *Arabidopsis* was analyzed using packages for R-statistical language and using an online microarray resource, *Genevestigator* (v 3.0) (Zimmermann et al., 2004). Microarray datasets from studies done by different labs and using platforms were difficult to process through our *in-house* scripts, primarily due to the batch-effects, and differences in numbers and identifiers of probe-sets corresponding to different platforms. Such datasets were analyzed through *Genevestigator* tool (Hruz et al., 2008); this study is referred to as “*Genevestigator*” analysis from here on. Independent to the *Genevestigator* analysis, the comprehensive microarray datasets from “AtGenExpress” study were processed and analyzed through *in-house* R-language scripts; this is referred to as “stand-alone” analysis from here on. Please see Supplementary Text Presentation 1 for more information on both the *Genevestigator* and stand-alone analysis.

Genes that preferentially up-regulate upon nematode infection were identified using *Genevestigator*; stand-alone analysis to identify such nematode-responsive genes could not be performed because the microarray datasets were not available in public domain at that time this study was performed. *Genevestigator* (v3) classified the microarray data from nematode infection assays into two stages—“early” and “late” based on days post infection. Probe sets that showed an up-regulation (>1.5-fold-change, log2-scale) in both “early” and “late” stages were downloaded in tab-separated file format and used for downstream analysis. On other hand, genes that show root-specific expression were identified using both the *Genevestigator* and stand-alone analysis. In stand-alone analysis, probe sets that showed a consistent up-regulation (>2-fold-change, log2) across all three contrasts, including with vegetative stem, inflorescence and leaf samples, at each stage were recorded (see Supplementary Text Presentation 1); this process was repeated for three time points (7, 17, and 21 days). Since, “AtGenExpress” data represented select developmental stages, we collected probe sets that are preferentially expressed (>1.5-fold-change, log2-scale) in root samples (six root zones) compared to other tissues or plant samples from all datasets (except AtGenExpress) available in *Genevestigator* (v3).

### Motif discovery using MEME

Multiple EM for Motif Elicitation (MEME) was used for the discovery of novel putative motifs. A third-order Markov chain background model was prepared from the upstream sequence of all the *Arabidopsis* genes. The upstream sequences for 27,144 genes were extracted using RSAT (Thomas-Chollier et al., 2008). Repetitive DNA elements were masked from the input sequences using Repeat Masker program (Smyth, 2004). Two different modes, *Any Number of Repetitions* (ANR) and *Zero or One Occurrence per Sequence* (ZOOPS) were used with the following parameters:

-nmotifs 30 -minsites 4 -maxsites 12 -minw 6 -maxw 12 -revcomp -dna -mod anr–bfile

Where, number of -minsites and -maxsites is given based on number of input sequences and the third order background model is provided for–bfile.

-nmotifs 30 -minw 6 -maxw 8 -revcomp -dna -mod zoops–bfile

Where, -minw 6–10 and -maxw 8–12 and the third order background model is provided for –bfile.

For ANR mode, we considered 30 motifs ranked by their significance levels of length between 6 and 12 nt, while for ZOOPS mode we had three runs, each predicting 10 statistically ranked motifs corresponding to motif length of 6–8 nt, 8–10 nt, and 10–12 nt, respectively. The statistically-significant motifs were screened for similarity with known transcription factor binding motifs using POXO (Kankainen et al., 2006) (http://ekhidna.biocenter.helsinki.fi/poxo) and STAMP (Mahony and Benos, 2007).

### Identification of known, conserved cis-regulatory elements from NRRS gene promoters

Promoters of NRRS genes identified along with the previously reported genes as positive control (*TobRB7, Atcel1, Hahsp17*, and *Lemmi9*) were subjected to comparative analysis for identification of conserved *cis*-elements. The upstream region from the start codon was extracted to include only the intergenic region (~1.5 kb) using Regulatory Sequence Analysis Tool (RSAT) (http://www.rsat.eu/) (Thomas-Chollier et al., 2008). The known *cis*-elements present in each of these promoters were collected using two different public resources, PLACE (Higo et al., 1999) and AT*cis*DB (Davuluri et al., 2003). The set of reported motifs were further used to elucidate conserved *cis*-elements using a Python script.

### Expression analysis of putative NRRS genes

Total RNA was isolated from *M. incognita*-infested galls and whole roots of *Arabidopsis* using RNeasy mini kit (Qiagen, Germany) following the manufacturer's protocol. The RNA integrity was checked by formaldehyde gel electrophoresis with ethidium bromide dye. Nanodrop spectrophotometer 8000 (Thermo Scientific, USA) was used to calculate purity ratios and quantify total RNA. cDNA was prepared using Protoscript M-MuLV first strand synthesis kit (NEB, USA) using oligo-d (T)23VN primers. cDNA was normalized and re-quantified before qRT-PCR. RT Primers (Supplementary Table 1) were designed from the cDNA sequence of selected genes using “Primer3” portal (http://frodo.wi.mit.edu/primer3/) (Rozen and Skaletsky, 2000).

The cDNA from infected and control samples was quantified and used as template with three biological replicates along with three technical replicates for each biological replicate. To ensure purity of the master mix and reaction mix setup, a non-template control reaction was included in every plate. A 20 μl reaction volume consisting of SYBR FAST qRT-PCR Master Mix (2x) Universal (KAPA Biosystems) and 10 pmol of each primer was used in all qRT-PCR reactions. The qRT-PCR reactions were performed on a StepOnePlus™ Real-Time PCR system with the following cyclic conditions: initial denaturation temperature of 95°C for 10 min followed by 40 cycles of 95°C for 15 s and 61°C for 45 s. The PCR products were exposed to melting curve analysis; the conditions were incubation at 60–95°C with a temperature increment of 0.3°C s^−1^ (Applied Biosystems®). The threshold cycle values were normalized by plant *UBQ10* (Supplementary Table 1) as endogenous control and fold changes of the target gene were calculated by 2^−ΔΔCt^ method (Livak and Schmittgen, 2001).

### Preparation of promoter::GUS construct

The promoter regions (1.5 kb upstream of the start codon) of NRRS genes, *At1g74770* and *At2g18140*, were amplified from the *Arabidopsis* genomic DNA using the gene specific oligonucleotides (Supplementary Table 1), with flanking restriction sites, *Bam*HI and *Sal*I. The amplified products were eluted from gel using Pure Link Gel Extraction kit (Invitrogen, USA), quantified and digested with *Bam*HI and *Sal*I. The 1.5 kb *Bam*HI/*Sal*I promoter fragment was cloned upstream of the GUS gene, using T4 DNA ligase (NEB, USA), with linearized *Bam*HI*/Sal*I digested pORE-R2 (Coutu et al., 2007) vector and transformed to *DH5-*α strain of *E. coli* (NEB, Massachusetts, USA). The prm::GUS fusion constructs were validated by nucleotide sequencing and introduced into *Agrobacterium tumefaciens* (Smith and Townsend, 1907) strain GV3101. *Arabidopsis* plants were transformed using the floral dip method (Clough and Bent, 1998). The primary transformants were selected on medium containing kanamycin (50 μg/ml) and further grown to develop T_3_ seeds. For each promoter, five independent *Arabidopsis* transgenic lines were tested for their response to nematode infection.

### GUS assay

Histochemical localization of GUS activity was performed with the substrate 5-bromo-4-chloro-3-indolyl-β-D glucuronide (X-Gluc) (Jefferson, 1989). The infected plants were uprooted and washed with water until roots were free of soil. The soil-free plants were immersed in freshly prepared GUS assay buffer [0.5 mM X-Gluc, 0.1 M NaHPO_4_ pH 8.0, 0.5 mM K_3_Fe(CN)_6_, 0.5 mM K_4_Fe(CN)_6_, 0.01 M EDTA pH 8.0, 20% methanol, and 0.1% Triton X-100] and vacuum infiltrated for 5–10 min in a desiccator. The tubes were then incubated overnight in dark at 37°C. The tissues were cleared by replacing the buffer with 70% ethanol and then imaged with a stereomicroscope (Nikon®, Japan) with an external fiber optic light source. The plants were monitored for GUS activity at 10 and 21 days after nematode infection.

### Cloning of At1g74770 promoter in pBC-06 RNAi vector

A 349-bp *splicing factor* sequence (AW828516) was amplified from *M. incognita* and cloned in pBC06 RNAi vector in sense and anti-sense directions (Yadav et al., 2006). CaMV35 promoter was removed from above vector and replaced with At1g74770 root-specific promoter using *Sbf* I and *Bam*HI restriction enzymes. All cloning steps were performed according to the protocols described by Sambrook et al. (1989), and the constructs were confirmed by restriction fragment analysis and sequencing. The binary vectors were transferred to *A. tumefaciens* strain GV3101 by freeze and thaw method (Weigel and Glazebrook, 2006). *A. thaliana* (*Col*-0) plants were transformed with *pAt1g74770::splicing factor* and an empty pBC-06 vector through floral dip method (Jefferson, 1989). The transformed plants were selected on kanamycin (50 μg/ml). T_2_ transgenic plants were raised and confirmed through PCR analysis (data not given). Five independent transgenic lines were developed and two transgenic lines were evaluated against *M. incognita* infection. Nematode infection assays were performed in T_3_ plants and the numbers of galls were calculated.

For gene expression study, RNA isolated from dsRNA expressing transgenic plant and control plant used as template, with setup of three biological replicates along with three technical replicates for each sample. To ensure purity of the master mix and reaction mix setup, a non-template control was included in every plate. A 20 μl reaction volume consisting of SYBR FAST qRT-PCR Master Mix (2x) Universal (KAPA Biosystems) and 10 pmol of each primer was used in all qRT-PCR reactions. The qRT-PCR reactions were performed on a StepOnePlus™ Real-Time PCR system with the following cyclic conditions: initial heating temperature of 95°C for 10 min followed by 40 cycles of 95°C for 15 s and 61°C for 45 s. The PCR products were exposed to melting curve analysis; the conditions were incubation at 60–95°C with a temperature increment of 0.3°C s^−1^ (Applied Biosystems®). The threshold cycle values were normalized by nematode 18S ribosomal RNA (Supplementary Table 1) as endogenous control and fold changes of the target gene were calculated by 2^−ΔΔCt^ method (Livak and Schmittgen, 2001).

## Results

### Identification and selection of nematode-responsive and root specific (NRRS) candidate genes

Candidate NRRS genes were identified in three steps: (a) an analyses of microarray data was performed to find genes that were induced by nematode infection and genes with root-specific expression patterns, (b) these root-specific and nematode-responsive gene sets were collated to find genes with desired NRRS gene expression pattern, and (c) finally, a *cis*-element based screening of candidate NRRS gene promoters was performed to select final candidates for downstream analyses.

**Figure 1:**
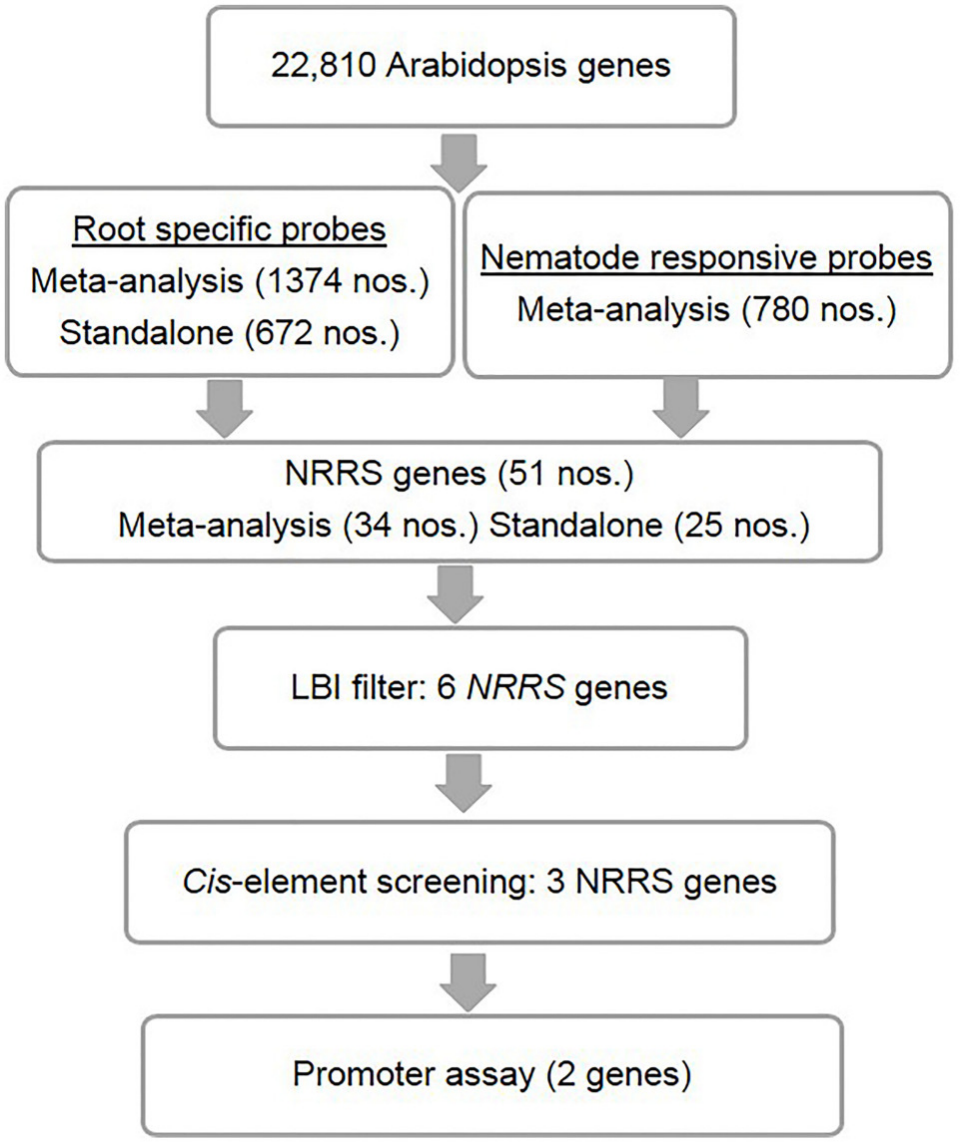
Identification of nematode-responsive root-specific (NRRS) genes. The top level consists of *A. thaliana* root-specific probes identified through both meta-analysis and stand-alone analysis. However, *M. incognita* nematode-responsive probes were identified through meta-analysis alone. Below that are the genes that were the result of cross-comparison with nematode-responsive genes and root-specific genes sets. After identifying 51 NRRS genes, an LBI filter was imposed which reduced the number of genes to six with low basal expression under control conditions. Further screening, based on *cis*-elements, led to identification of three NRRS genes. The lowest level includes the final two LBI genes that have been identified based on the results of screening by experimentally validated *cis*-elements, identified in earlier studies.

**Figure 2:**
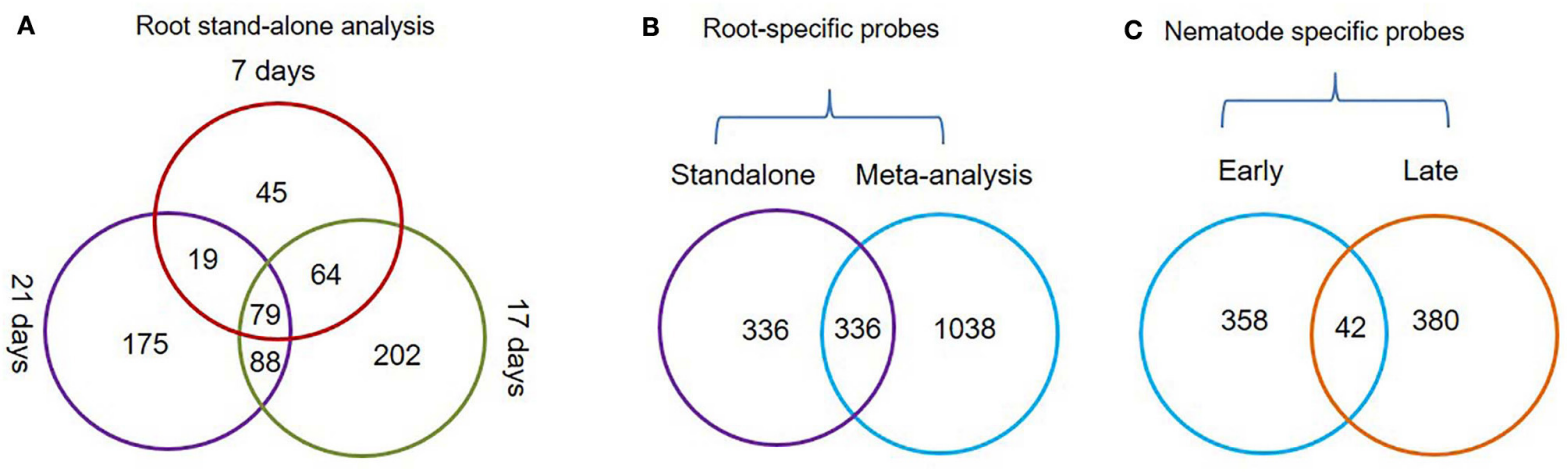
Venn diagrams depicting the status of root-specific and nematode-responsive probes. **(A)** Probes differentially expressed in the root at three different time points (7, 17, and 21 days) and a subset of common probes (79 nos.) constantly up-regulated in roots using the stand-alone studies. (**B)** Probes that were identified as root-specific by both meta-analysis and stand-alone analysis. **(C)** Probes that were constantly up-regulated during early and late stages of *M. incognita* nematode infection.

The *Genevestigator* analysis i.e., meta-analysis of microarray datasets identified 1,374 probe sets that showed statistically significant and consistent up-regulation (>1.5-fold-change, log2 scale) in different (*n* = 6) root zones (Figure 1). On other hand, the stand-alone analysis, comparing root samples with vegetative shoot, inflorescence and leaf samples identified a total of 672 probe sets from three developmental time points (7, 17, and 21 days). In process, we also identified, 79 probe sets that were consistently enriched in root across all three time points investigated in stand-alone analysis (Figure 2A, Supplementary Table 2) and 336 probe sets that were common between *Genevestigator* and stand-alone analysis (Figure 2B). The low overlap between probes sets from *Genevestigator* and stand-alone analysis could be because of the additional data processing and summarization steps that *Genevestigator* performs to reduce batch effects and to provide robust expression estimates from different microarray platforms. In addition to root-specific probe sets, the *Genevestigator* analysis for nematode-induced genes (>1.5-fold-change, log2 scale) from “early” and “late” stages of nematode infection yielded 780 probe sets, with 42 probe sets showing a constant up-regulation in both stages (Figure 2C, Supplementary Table 3).

These probe sets were converted to *Arabidopsis* Gene Identifiers (AGIs) and collated to identify candidate NRRS genes. If multiple probes represented a single gene then the probe with highest median expression across samples was selected to represent the gene, and if a single probe represented multiple genes then all of these were included in final set. This conversion resulted in 700 root-specific AGIs from stand-alone analysis, 1,452 root-specific AGIs from meta-analysis and 850 nematode-responsive AGIs (Table 1). The nematode-responsive (NR) AGIs (*n* = 700) were then compared with root-specific (RS) AGIs from both the *Genevestigator* analysis (*n* = 1,452) and the stand-alone analysis (*n* = 700) to identify genes with nematode responsive and root-specific expression (*n* = 51). We refer to these 51 genes as candidate NRRS genes; these included 25 and 34 genes from NR vs. RS comparisons, where RS genes corresponded to stand-alone (Supplementary Table 4) and *Genevestigator* analysis (Supplementary Table 5) respectively. Both these categories NR vs. RS from stand-alone and NR vs. RS from *Genevestigator* analysis has eight common genes (Supplementary Table 6). Finally, to select genes that have a low expression under normal developmental stages, which is a desired characteristic for gene promoters that will drive expression of RNAi construct, we filtered genes based on average expression values in root tissues (see Supplementary Text Presentation 1). Six genes passed the low basal intensity (LBI) filter and were considered for next step i.e., a *cis*-element based screening (Table 2).

**Table 1:** Low Basal Intensity genes (in absence of nematode) from meta-analysis and stand-alone analysis.

**Comparisons**	**Root-specific *Arabidopsis* gene identifiers (nos.)**	**Nematode-responsive *Arabidopsis* gene identifiers (nos.)**	**Predicted “low basal intensity” *Arabidopsis* gene identifiers (nos.)**	**Common *Arabidopsis* gene identifiers between both comparisons**
Root-specific (standalone) vs. nematode-responsive	700	850	25	8
Root-specific (meta-analysis) vs. nematode-responsive	1,452	850	34	

**Table 2:** Summary of the genes (*n* = 6) qualifying the Low Basal Intensity filter along with other details.

***Arabidopsis* gene identifier**	**Gene name**	**Annotation**	***M. incognita* infected (early) Log 2 ratio**	***M. incognita* infected (late) Log 2 ratio**	**% of expression signal in roots**
At1g74770	BTSL1	Zinc ion binding protein	2.48	0.79	94
At1g80320		2-Oxoglutarate (2OG)	1.74	0.29	60
At1g48670		Auxin-responsive GH3family protein	1.6	0.49	65
At3g29775		Transposable element gene	1.18	1.52	29
At5g58780	ATCPT5	Heptaprenyl diphosphate synthase	3.22	−0.03	74
At2g18140		Peroxidase superfamily	1.7	1.26	88.5

### Conserved *Cis*-regulatory elements in the promoters of nematode-responsive and NRRS gene

**Figure 3:**
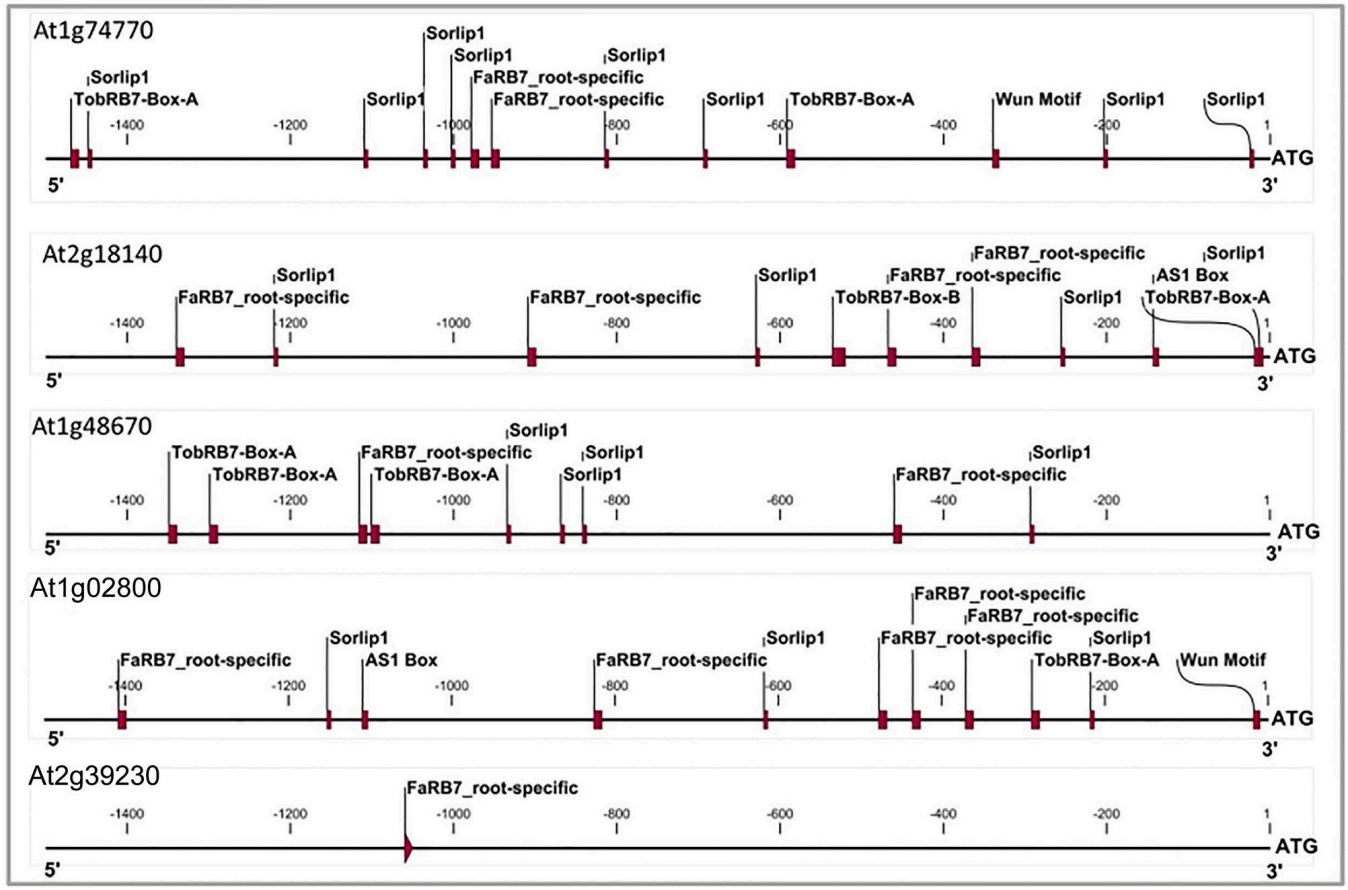
Cis-elements profile of promoters of three genes which qualified NRRS genes screening along with At1g02800 gene (AtCel2 positive) and At2g39230 gene (LOJ gene- lateral organ Junction gene and considered as negative gene unlikely to have any role in root development and nematode responsiveness). The TobRB7-Box-A/Box-B elements are nematode-responsive and Sorlip1, AS1 box and FaRB7 are root-specific elements. Please note overabundance of NRRS elements in NRRS genes and AtCel2 gene as compared to gene unlikely to be involved in nematode responsiveness and root development. The 1.5 kb region of each promoter was used for the profiling.

The six candidate NRRS genes were screened for the presence of *cis-*regulatory elements (Table 3) that have been experimentally validated to play a role in root-specificity i.e., *AS1*, Sorlip1, and FaRB7, and typical for promoters of genes that are upregulated in nematode feeding site (NFS) upon infection i.e., *TobRB7* box A/B etc. The purpose of this regulatory element based screening was to further reduce the number of genes selected for downstream molecular cloning and *in planta* validation. Out of six NRRS genes, only three genes—*At1g74770, At2g18140*, and *At1g48670* (Figure 3) included regulatory elements from both the nematode responsive and root specific (NRRS) categories in their promoter regions. *ATCEL2*, earlier implicated in nematode-responsiveness as well as root specificity used as positive control (Wieczorek et al., 2008). Gene *At2g39230*, coding for a LOJ protein (Saha et al., 2007) served as negative control and had no nematode-responsive as well as root-specific elements except for single FaRB7 element. Two genes, *At5g58780* and *At3g29775* lacked root-specific elements whereas the *At1g80320* gene promoter contained only the E-box motif out of all the studied elements (*n* = 12). Surprisingly, except for the E-Box motif, all other putative motifs described by Sukno et al. (2006) were found to be distributed in all three categories of genes, TobRB7, *Atcel1, Hahsp17*, and *Lemmi9* that show elevated expression upon nematode infection in the NFS (Opperman et al., 1994; Escobar et al., 1999, 2003; Sukno et al., 2006), genes that are downregulated in NFS—*AtPAl1, AtTIP*, and *AtANT1* (Hammes et al., 2005) and *UBP22/At5g10790* that does not show any significant difference in expression pattern in response to nematode infection (Favery et al., 2004). Therefore, these putative motifs (W-Box, ElRE, ERE, and P-Box) proposed by were not included in this step to filter NRRS gene based on presence of cis-regulatory elements (Table 3).

**Table 3:** Nematode-responsive (NR) and Root-specific (RS) *cis*-elements used for screening of 6 NRRS genes passing Low Basal Intensity filter.

***Cis*-element**	**Consensus sequence**	**Context**	**References**
E-BOX	CAATTG	Binds to nuclear proteins from galls, nematode-responsive element	Escobar et al., 1999; Puzio et al., 2000
EIRE	TTCGacc	Elicitor responsive element core	Puzio et al., 2000; Mitchum et al., 2004
ERE	ATTTCaaa	Elicitation, wounding and pathogen	Sukno et al., 2006
P-Box	CCTTtg	Conserved among nematode-responsive genes as well	Sukno et al., 2006
WUN-Motif	aAATTtcct	Wound-responsive	Puzio et al., 2000
TobRB7-Box-A	CGAGCTCGNNA	Root-specific and nematode-responsive	Yamamoto et al., 1991; Opperman et al., 1994; Mitchum et al., 2004
TobRB7-Box-B	CAAAATGTGTTATTTTT	Root-specific and Nematode- responsive	
AS1-Box	TGACGTCA	Root-specific *cis*-element	Puzio et al., 2000
*Oryza sativa*_Root-specific	gGTACGTGGCG	ABA responsive and root-specific *cis*-element	Ono et al., 1996
FaRB7_root-specific	TTTCNTTTTGG	Conserved motif in root-specific genes	Vaughan et al., 2006
W-Box	TTGACT	*Cis*-element essential for elicitor responsiveness	Yu et al., 2001; Thurau et al., 2003
Sorlip1	AGCCAC	Over-represented in root-specific genes	Jiao et al., 2005

**Figure 4:**
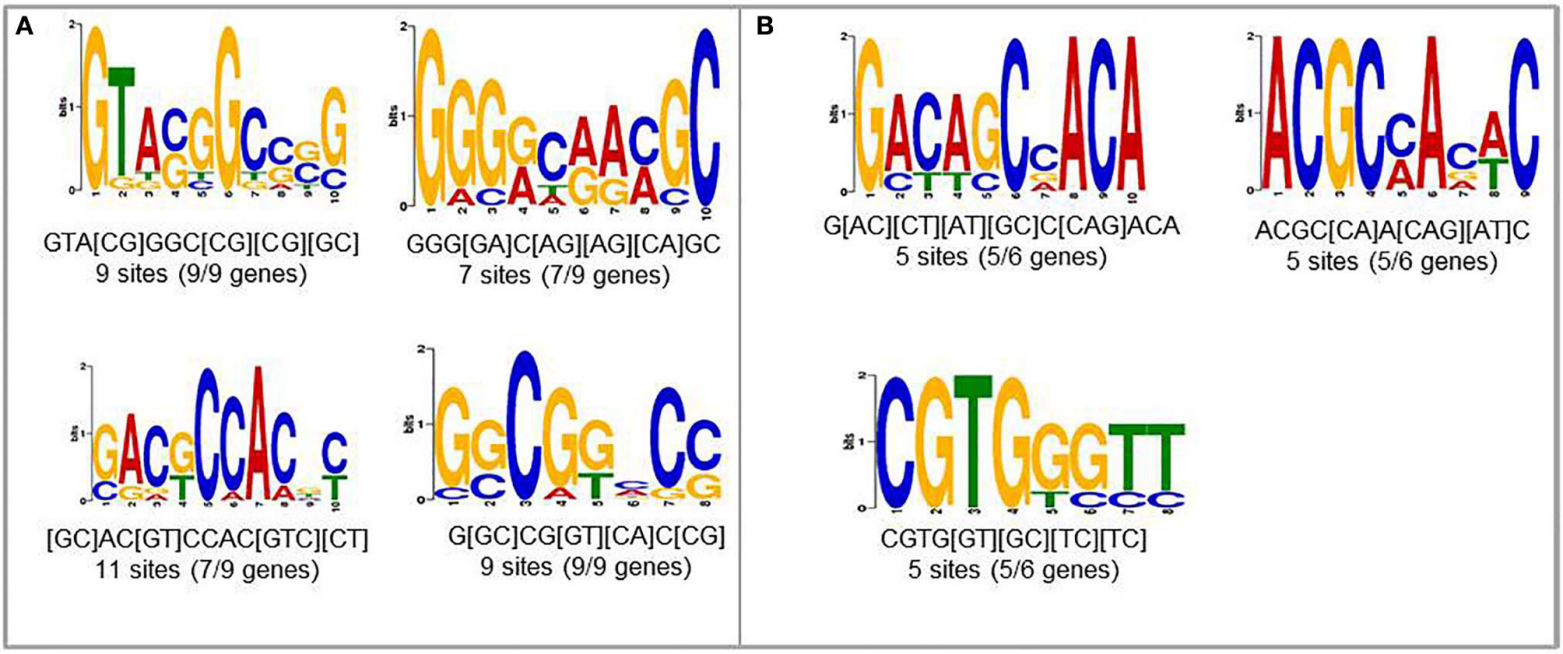
Novel *cis*-elements predicted using MEME tool. **(A)** Input set containing both experimentally validated (earlier published) and predicted (present study) NRRS promoters. **(B)** Novel NRRS *cis*-elements predicted from set of NRRS promoters identified in this study.

To find new candidates that may play role in upregulation of nematode-responsive genes in NFS, we used promoters from two different set of genes. First set consisted of genes that are well-studied for role in establishing compatible plant nematode interaction and show elevated expression upon infection (*Lemmi9, TobRB7*, and *Hahsp17*) along with the six NRRS genes predicted in this study (*At1g74770, At1g48670, At2g18140, At3g29775, At5g58780*, and *At1g80320*). The second set consisted just the six NRRS gene promoters from this study. We identified four motifs which were common between promoters of well-studied nematode-responsive genes and our NRRS genes (Figure 4A). In addition, we found three new motifs that are present only in NRRS genes (Figure 4B). Later on, MEME analysis has been carried out for the promoter region of all 51 genes to identify NRRS motif (Supplementary Figure Presentation 1; Supplementary Figure 1). To identify the transcription factors that might bind to these sites, we used POXO (Kankainen et al., 2006) and STAMP (Mahony and Benos, 2007) but no statistically significant similarity to known motifs was found. Therefore, further characterization of these novel motifs is required to establish their functional roles in NRRS activity.

### Confirmation of nematode-responsive expression pattern of NRRS genes

**Figure 5:**
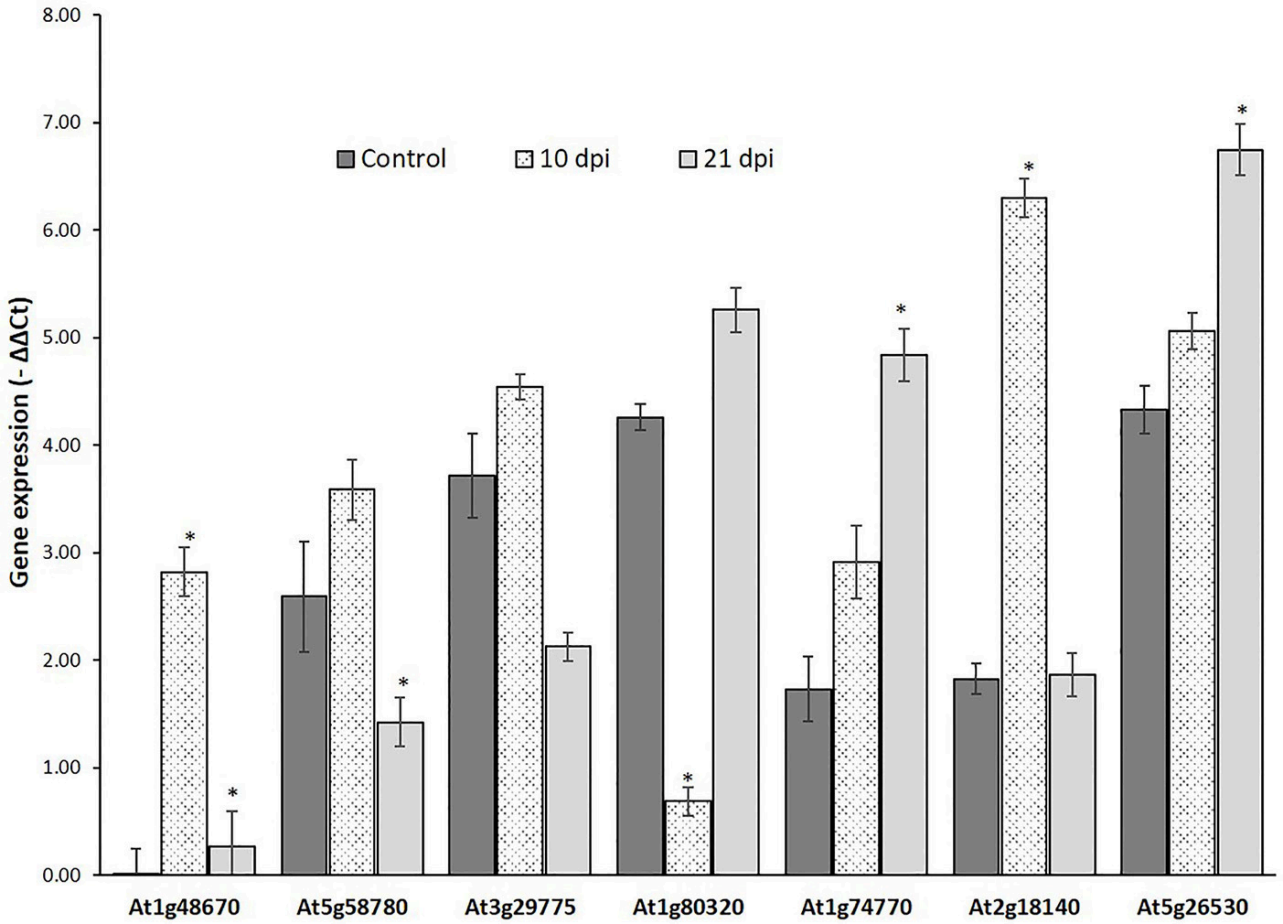
Expression analysis of nematode-responsive root-specific genes using Quantitative Real time PCR. Relative expression of six NRRS genes (At1g48670, At5g58780, At3g29775, At1g80320, At1g74770, and At2g18140) along with At5g26530, a nematode-responsive root-specific gene used as positive control (Kumar et al. 2016) in control and nematode infected plant root samples at 10- and 21- dpi. The transcripts levels were normalized to the expression of a plant *UBQ10* gene. The data are shown as −ΔΔC_t_ and each bar represents the mean ± SE (*n* = 3). An asterisk indicates statistical significance difference in a one-way ANOVA and Tukey test (*p* ≤ 0.05).

Genes that passed the cis-regulatory element based criteria were evaluated for expression patterns in response to nematode infection before proceeding for molecular cloning their promoter regions. First, the expression patterns of six genes, *UBP7, OXA1, Actin, RPN7, ATPase* (Jammes et al., 2005), and *UBQ10* (accession number DQ793132.1) was investigated in response to nematode infection for selection of appropriate internal controls. *UBQ10* gene showed a consistent expression in both wild-type and nematode infected plants and was used as an internal controls for quantitative real time (qRT) PCR assay. The expression of six NRRS genes—*At1g74770, At2g18140, At1g80320, At1g48670, At5g58780*, and At3g29775 was studied at two different time points (10 and 21 dpi) along with At5g26530 gene, earlier implicated in nematode-responsiveness, which served as positive control (Kumar et al., 2016). The relative expression of At2g18140 and At1g74770 was maximum at 10 and 21 dpi, respectively as compared to control (Figure 5).

### *In Vivo* validation of NRRS promoters

**Figure 6:**
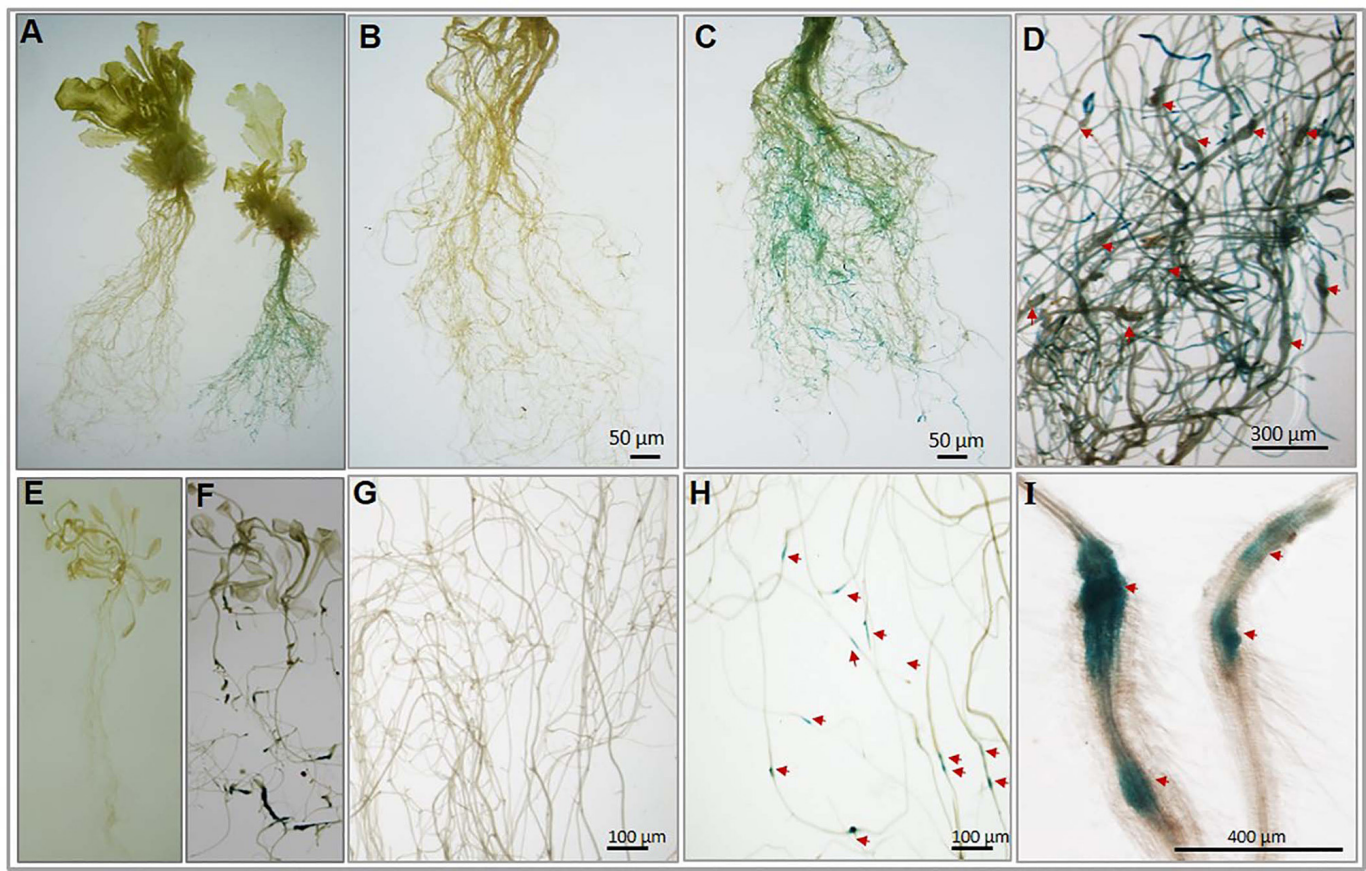
Histochemical GUS analysis of transgenic *Arabidopsis* lines harboring nematode-responsive root-specific promoters. **(A)** Control plant (w/o nematode infection) plant (left) and nematode infected plant (right) at 21 dpi. **(B)** Control (w/o nematode infection) root of transgenic (pAt1g74770::GUS) plant. **(C)** Nematode infected root of transgenic (pAt1g74770::GUS) plant exhibiting strong GUS activity throughout the root at 21 dpi. **(D)** Enlarged microscopic view of nematode infected root of transgenic (pAt1g74770::GUS) plant exhibiting strong GUS activity and multiple galls at 21 dpi. **(E)** Control (w/o nematode infection) transgenic plant (pAt2g18140::GUS). **(F)** Nematode infected transgenic plant (pAt2g18140::GUS) showing GUS activity at 10 dpi. **(G)** Control (w/o nematode infection) root of transgenic (pAt2g18140::GUS) plant. **(H)** Nematode infected root of transgenic (pAt2g18140::GUS) plant exhibiting GUS activity only in the nematode induced galls at 10 dpi. **(I)** Enlarged microscopic view of galls from nematode infected roots of transgenic (pAt2g18140::GUS) plant showing GUS activity at 10 dpi. The red arrow in **(D,H)** and **(I)** point to the nematode galls.

To experimentally confirm the root-specific and nematode-responsive behavior of the shortlisted genes, we generated *promoter::GUS* constructs for both the putative NRRS genes. Five independent transgenic lines for each promoter construct and 15 plants from each independent line were evaluated. In transgenic plants harboring p*At1g74770*::*GUS* constructs, the nematode infected plants (T3-P6) showed strong GUS expression in roots at 21 dpi (Figure 6A complete plant right side and Figure 6C) as compared to uninfected (control) plants (Figure 6A complete plant left side and Figure 6B). On examination of p*At1g74770*::*GUS* lines under microscope, large numbers of galls were visible along with blue staining throughout the root system (Figure 6D). In transgenic plants harboring p*At2g18140*::*GUS* constructs, the nematode infected transgenic line (T3-P2) revealed maximum GUS activity only in galls during early stages of infection at 10 dpi (Figures 6F,G) as compared to uninfected (control) plants (Figures 6E,H). The microscopic examination revealed strong GUS activity in the gall (Figure 6I). Thus, the histochemical GUS assay of promoter-reporter gene constructs upon nematode infection confirmed the root-specificity and nematode-responsiveness of both the promoters. GUS activity was not detected in control (uninfected) transgenic plants for either of the promoters used.

### Screening of p*At1g74770*::*splicing factor* RNAi transgenic lines for nematode resistance

**Figure 7:**
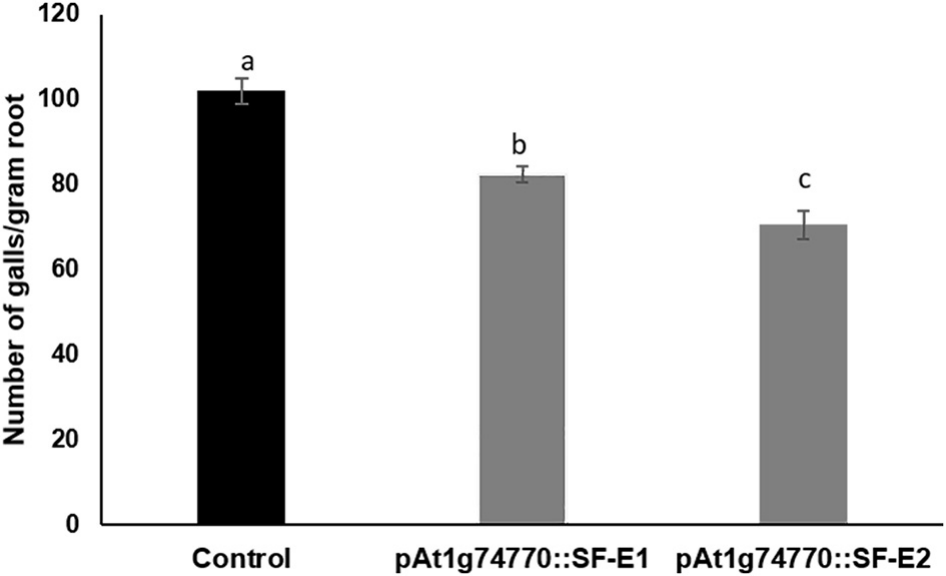
Root-knot nematode infection assay in control (empty vector) and transgenic plants expressing dsRNA of *splicing factor* gene using *At1g74770* promoter. Fifteen plants each of control and transgenic lines (two independent events, E1 and E2) were evaluated and the values shown are average number of knots per plant. Each bar denotes the mean ± SE (*n* = 15), and bars with different letters (a–c) indicate statistical significance difference in a one-way ANOVA (*p* ≤ 0.05).

**Figure 8:**
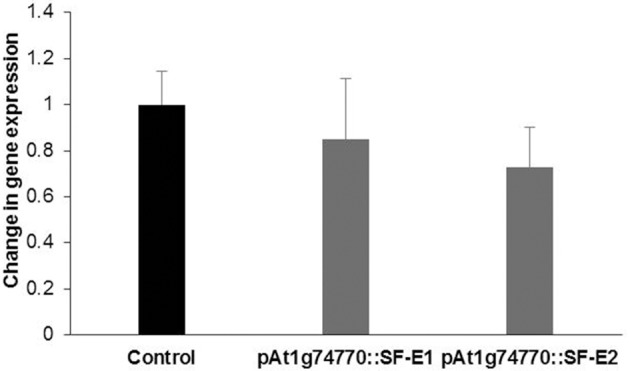
Quantitative Real-time PCR expression analysis of *splicing factor* gene. Expression levels of *splicing factor* gene in nematode females, developed in the dsRNA expressing *Arabidopsis* transgenic lines (pAt1g74770::SF-E1 and pAt1g74770::SF-E1) compared to control. The transcripts levels were normalized to the expression of a nematode *18S rRNA* gene. The data are shown as fold change and each bar represents the mean ± SE (*n* = 3). There was no statistical difference in data sets.

NRRS promoter (*At1g74770*) was used for expressing dsRNA of a nematode gene, *splicing factor*, to evaluate the efficacy of NRRS promoters in inducing HD-RNAi mediated resistance in *Arabidopsis*. Of the five independent transgenic lines containing promoter *At1g74770:*:*splicing factor, two* transgenic lines *(At1g74770::SF E1* and *At1g74770::SF E2)* were tested against nematode. For each transgenic line 15 plants were evaluated. Transgenic lines of the *splicing factor* gene exhibited 20–32% reduction in number of galls compared to control plants (Figure 7). To study the effect of transgenic lines on gene expression in nematodes, females were isolated from transgenic lines expressing *splicing factor* dsRNA and control plants at 42 dpi. Quantitative Real-Time PCR (qRT-PCR) analysis revealed slightly reduced expression of *splicing factor* gene in the females isolated from transgenic plants compared to control, indicating that the *splicing factor* gene, driven by the NRRS promoter, has not been silenced significantly (Figure 8).

## Discussion

Plant parasitic nematodes (PPNs) are one of the major threat to crops across the globe, and RKN (*Meloidogyne* spp.) account for a major proportion of damage caused by nematodes (Elling, 2013). Among different biotechnological approaches to control nematode infestation, silencing of nematode genes crucial for primary infection and reproduction through RNAi has emerged an effective method (Dutta et al., 2015). However, this controlled targeting of nematode genes is contingent upon expression of RNAi constructs in response to nematode infection, therefore depends upon the identification of NRRS genes. In this study, we identify such gene using *in silico* analysis of available microarray data and demonstrate the proof-of-concept for host-delivered, RNAi-mediated nematode resistance in *Arabidopsis*.

### Nematode-responsive root-specific (NRRS) genes

The NRRS gene set identified in this study comprises of several putative nematode responsive and root specific genes such as those from family of expansins, peroxidases, and PINFORMED auxin transporter, that have well-established roles in plant–nematode interactions. Expansins facilitate nematode penetration into the roots (Gal et al., 2006; Fudali et al., 2008); up-regulation of expansins and expansin-like genes are reported in developing galls in *Arabidopsis* (Jammes et al., 2005), tomato (Gal et al., 2006) and in soybean syncytia induced by soybean cyst nematodes (Ithal et al., 2007). Peroxidases play an essential role in cell wall strengthening and are also reported to be up-regulated in nematode-infected roots (Ithal et al., 2007). The up-regulation of both expansins as well as peroxidases is proposed to be part of mechanism that maintains a balance between cell wall loosening and cell wall strengthening during feeding site development of nematodes (Gheysen and Mitchum, 2009). *PIN* are efflux facilitators that mediate auxin transport; these express in specific regions of roots and show elevated expression upon nematode infection (Grunewald et al., 2009). The presence of these gene members of families that have established roles in mediating successful plant nematode interactions such as AT*EXPA14*, ATEXP6, ATEXP4, ATEXPB3, and ATAXP4 in nematode-responsive gene set along with AT*CYP86* (a peroxidase), ATRFNR2 in NRRS gene set shows the effectiveness of such strategy to identify tissue-specific and pathogen responsive genes. In addition to, the presence of *cis-*regulatory elements, other than those used for filtering NRRS genes, such as those that play role wounding, pathogen response, plant defense signaling, disease, and pathogen responses and most importantly, the elements involved in organ specificity (Table 3) further supports that validity of approach used for selection of NRRS genes. Thereby, demonstrating that expression-based surveys for identifying condition-specific gene promoters may have a potential utility in developing plants with host-mediated response to biotic and abiotic response.

### Nematode-responsive root-specific promoter driven expression upon nematode infection

*In planta prm::GUS* constructs of both NRRS genes tested in this study (*At1g74770* and *At2g18140*) demonstrates highly restricted expression in roots in response to nematode infection. The *At2g18140*, expression is highly restricted to galls that encapsulates NFS with no trace in aerial portion of plant. *At2g18140* encodes for a peroxidase protein and plant peroxidases have earlier been implicated in host-plant parasitic nematode interaction (Vercauteren et al., 2001; Jammes et al., 2005; Severino et al., 2012), including *Coffea canephora* sp. Thereby, confirming important and likely conserved roles of peroxidases in response to PPNs. The *At1g74770* gene promoter displays *GUS* expression throughout the root, strong and constitutively present across all cell layers. Thereby, it seems like an ideal candidate to develop nematode-resistant plants.

### Nematode infection assay of p*At1g74770*::*splicing factor* transgenic plant

The number of galls in *Arabidopsis* transgenic plants expressing p*At1g74770*::*splicing factor* gene was lower (20–32%) compared to control plants. However, transgenic tobacco plants expressing dsRNA of *splicing factor* gene through CaMV35S promoter exhibited about 95% reduction in gall formation as well as in number of nematode females (Yadav et al., 2006). Similarly, the *splicing factor* gene driven by 35S promoter in *Arabidopsis* transgenic lines exhibited up to 71% reduction in gall number (Kumar et al., 2017). In the last few years, several promoters have been identified in various crops including tobacco (Opperman et al., 1994), *LEMMI9* in tomato (Escobar et al., 1999), *Hahsp17.7G4* in *Helianthus annuus* (Escobar et al., 2003), *AtCel-1* in *Arabidopsis* (Sukno et al., 2006), *AtWRKY23* (Grunewald et al., 2008), and *ZmRCP-1* in maize, banana, and plantains (Onyango et al., 2016). None of the promoters identified have been utilized for driving the production of dsRNA of nematode genes for HD-RNAi silencing. Only TobRB7, a gall specific promoter was used to drive *M. javanica* gene, MjTis11, in tobacco but no sign of gall reduction was observed in nematode infected plants (Fairbairn et al., 2007). The lack of silencing observed in the TobRB7 promoter lines was attributed to the weakness of the TobRB7 promoter. However, we observed up to 32% reduction in nematode infection by using our NRRS promoter as compared to 71% reduction with use of CaMV35S promoter. The CaMV35S promoter, being constitutive in nature is likely to produce more dsRNA as compared to the conditional NRRS promoter used in the present study. However, the use of 35S promoter has to be exercised with caution since dsRNA is produced in all tissues all the time and can lead to undesirable effects in transgenic plants. There is a need to identify and evaluate large number of tissue-specific promoters and use the best ones for developing nematode resistant plants using HD-RNAi approach.

## Conclusion

Plant-parasitic nematodes (PPNs) are primary biotic factors that limit crop production. RNA interference (RNAi) presents a practical approach for silencing of multiple nematode parasitism and developmental genes via the host-mediated response. This study presents a computational approach to identify NRRS gene promoters and demonstrates its practical utility for host-induced RNAi-mediated control of nematode infestation in *Arabidopsis* as a proof-of-concept. Although further work is required to improve the efficiency of nematode control by testing other nematode gene targets, the study provides a general framework that addresses concerns regarding the use of constitutive promoters that may lead to off-targets effects and represent one more step toward the development of crops with a built-in defense mechanism against invading pathogens.

## Author contributions

AtK, AnK, AS, KS, RS, and PKJ planned the experiments. AtK designed and performed *in silico* analysis for NRRS promoters, cis-element identification. AtK performed molecular cloning of promoters and expression based validation. AnK generated nematode infected root samples, carried out gene expression studies through qRT-PCR, developed transgenic lines, carried out GUS analysis and RNAi construct preparation. AtK and AnK took the photographs. AtK, AnK, VS, AS, MA, RB, KS, RS, and PKJ analyzed the results. AtK, AnK, and PKJ wrote and finalized the manuscript. All authors read and approved the final manuscript.

### Conflict of interest statement

The authors declare that the research was conducted in the absence of any commercial or financial relationships that could be construed as a potential conflict of interest.

## Supplementary material

Supplementary Table 1 Primers used for qRT-PCR analysis and amplification of target promoter sequences from the *Arabidopsis* (Col 0) genomic DNA.

Supplementary Table 2 Probes preferentially expressed (FC ≥ 4 and corrected *p*-value ≤ 0.01) in roots among all three studied time points (7, 17 and 21 days).

Supplementary Table 3 Genes that were found to be up-regulated, in *Arabidopsis thaliana* on *Meloidogyne incognita* infection at the early and late stages, through meta-analysis.

Supplementary Table 4 NRRS genes identified using standalone analysis.

Supplementary Table 5 NRRS genes identified using meta-analysis.

Supplementary Table 6 NRRS genes (8 nos.) common between the standalone and meta-analysis.

Supplementary Text Presentation 1 Details of meta-analysis and stand-alone analysis.

Supplementary Figure Presentation 1 MEME analysis using promoter region of 51 nematode-responsive root-specific genes.

Supplementary Figure 1 MEME output for 51 NRRS genes for motif AAAxAAAA.

## Abbreviations

HD-RNAi: Host-delivered RNA interference
NRRS: Nematode-responsive root-specific
RKNs: Root-Knot Nematodes
PPNs: Plant Parasitic Nematodes
J2s: Second-stage Juveniles
DPI: Days Post Inoculation
LBI: Low Basal Intensity
G-MAI: Global-Mean of Average Intensities.
