# Supplementary material for: Identification, Validation and Utilization of Novel Nematode-Responsive Root-Specific Promoters in Arabidopsis for Inducing Host-Delivered RNAi Mediated Root-Knot Nematode Resistance
Source: Front Plant Sci. 2017 Dec 12;8:2049. doi: 10.3389/fpls.2017.02049 (PMC5733009; doi:10.3389/fpls.2017.02049)
Supplement: Supplementary Table 1 — Primers used for qRT-PCR analysis and amplification of target promoter sequences from the Arabidopsis (Col 0) genomic DNA. [file Table1.DOCX]

**Table 1 Primers used for qRT-PCR analysis and amplification of target promoter sequences from the *Arabidopsis* (Col 0) genomic DNA**.

| **Gene and Primer Code** | **Sequence (5´-3´)** |
| --- | --- |
| RT Primer for gene expression studies | |
| At1g74770 F | CAACTGCCAAACAGTTCAACAT |
| At1g74770 R | ACTCAAGTTCCTCCTGAAGCTG |
| At2g18140 F | TCAAGGCAGCATTAGAGAATGA |
| At2g18140 R | TCAGGAAGGTCTTTGTTTGGTT |
| AT1G48670 F | ATTTTCTTGAGAAACACTGGAAGG |
| AT1G48670 R | GCATTCTTGTTCAATAACATCAGC |
| AT1G80320 F | GAAACGATCCCTTCTGTCAAAC |
| AT1G80320 R | TCCTGTAACTCAGCATCCTCAA |
| AT3G29775 F | GGAGAAGAAACGTGGTTGGTAG |
| AT3G29775 R | ACACAACCTTTCCACCATTACC |
| AT5G58780 F | AGACATGTGGCAGTGATAATGG |
| AT5G58780 R | TTTAAAGCAAAGCTCGGAGAAC |
| AT1G26530 F | GTGTACTCAAAGGGACATATGCTG |
| AT1G26530 R | -TACATGATTGGTACACCAGGAATC |
| Plant Control UBQ-10F | GGAAAGACCATCACCCTTGA |
| Plant Control UBQ-10R | ATCCTCAAGCTGCTTTCCAG |
| Nematode 18S rRNA R | TCAACGTGCTTGTCCTACCCTGAA |
| Nematode 18S rRNA F | TGTGTACAAAGGGCAGGGACGTAA |
| Promoter cloning primer | |
| At1g74770 F | ACAGGCACATCAGCGGCACC |
| At1g74770 R | TTCCGGCGGAAGGGAATGAAG |
| At2g18140 F | TCTCGGATCCTTGCACATTAGGATAGGGCA |
| At2g18140 R | TTGTCGCTAGCTAGATCAAGAA |
