# Supplementary material for: Identification, Validation and Utilization of Novel Nematode-Responsive Root-Specific Promoters in Arabidopsis for Inducing Host-Delivered RNAi Mediated Root-Knot Nematode Resistance
Source: Front Plant Sci. 2017 Dec 12;8:2049. doi: 10.3389/fpls.2017.02049 (PMC5733009; doi:10.3389/fpls.2017.02049)
Supplement: Supplementary Table 2 — Probes preferentially expressed (FC ≥ 4 and corrected p-value ≤ 0.01) in roots among all three studied time points (7, 17 and 21 days). [file Table2.DOCX]

**S2 Table. Probes preferentially expressed (FC ≥ 4 and corrected *p*-value ≤ 0.01) in roots among all three studied time points (7, 17 and 21 days).**

| **S. No.** | ***Arabidopsis* Gene Identifiers** | **Probe** | **Gene Symbol** | **Gene Ontology Molecular Function** |
| --- | --- | --- | --- | --- |
|  | AT2G33790 | 267457_at |  |  |
|  | AT2G44010 | 267226_at |  |  |
|  | AT2G30840 | 267207_at |  | 0005506 // iron ion binding // inferred from electronic annotation /// 0009815 // 1-aminocyclopropane-1-carboxylate oxidase activity // inferred from sequence or structural similarity /// 0016491 // oxidoreductase activity // inferred from electronic annotation /// 0016702 // oxidoreductase activity, acting on single donors with incorporation of molecular oxygen, incorporation of two atoms of oxygen // inferred from electronic annotation |
|  | AT2G37750 | 267178_at |  |  |
|  | AT2G41480 | 267101_at |  | 0004601 // peroxidase activity // inferred from sequence or structural similarity /// 0004601 // peroxidase activity // inferred from electronic annotation /// 0005506 // iron ion binding // inferred from electronic annotation /// 0005509 // calcium ion binding // inferred from electronic annotation /// 0009055 // electron carrier activity // inferred from electronic annotation /// 0016491 // oxidoreductase activity // inferred from electronic annotation /// 0020037 // heme binding // inferred from electronic annotation /// 0046872 // metal ion binding // inferred from electronic annotation |
|  | AT2G39430 | 266978_at |  |  |
|  | AT2G25980 | 266838_at |  | 0005529 // sugar binding // inferred from electronic annotation |
|  | AT2G25810 | 266649_at | TIP4;1 | 0005215 // transporter activity // inferred from electronic annotation /// 0015250 // water channel activity // inferred from sequence or structural similarity |
|  | AT2G32020 | 265668_at |  | 0008080 // N-acetyltransferase activity // inferred from sequence or structural similarity /// 0008080 // N-acetyltransferase activity // inferred from electronic annotation /// 0016740 // transferase activity // inferred from electronic annotation |
|  | AT2G27370 | 265645_at |  |  |
|  | AT1G31885 /// AT2G21020 | 265435_s_at |  | 0005215 // transporter activity // inferred from electronic annotation |
|  | AT2G21045 | 265439_at |  |  |
|  | AT1G30870 | 265102_at |  | 0004601 // peroxidase activity // inferred from sequence or structural similarity /// 0004601 // peroxidase activity // inferred from electronic annotation /// 0005506 // iron ion binding // inferred from electronic annotation /// 0005509 // calcium ion binding // inferred from electronic annotation /// 0009055 // electron carrier activity // inferred from electronic annotation /// 0016491 // oxidoreductase activity // inferred from electronic annotation /// 0020037 // heme binding // inferred from electronic annotation /// 0046872 // metal ion binding // inferred from electronic annotation |
|  | AT1G52060 | 265049_at |  |  |
|  | AT1G67330 | 264998_at |  |  |
|  | AT1G05260 | 264577_at | RCI3 | 0004601 // peroxidase activity // inferred from direct assay /// 0004601 // peroxidase activity // inferred from sequence or structural similarity /// 0004601 // peroxidase activity // inferred from electronic annotation /// 0005506 // iron ion binding // inferred from electronic annotation /// 0005509 // calcium ion binding // inferred from electronic annotation /// 0009055 // electron carrier activity // inferred from electronic annotation /// 0016491 // oxidoreductase activity // inferred from electronic annotation /// 0020037 // heme binding // inferred from electronic annotation /// 0046872 // metal ion binding // inferred from electronic annotation |
|  | AT2G25160 | 264404_at | CYP82F1 | 0004497 // monooxygenase activity // inferred from electronic annotation /// 0005506 // iron ion binding // inferred from electronic annotation /// 0009055 // electron carrier activity // inferred from electronic annotation /// 0016491 // oxidoreductase activity // inferred from electronic annotation /// 0019825 // oxygen binding // inferred from sequence or structural similarity /// 0020037 // heme binding // inferred from electronic annotation /// 0046872 // metal ion binding // inferred from electronic annotation |
|  | AT2G28670 | 263437_at |  |  |
|  | AT2G36100 | 263284_at |  |  |
|  | AT1G30750 | 263227_at |  |  |
|  | AT2G16005 | 263098_at |  |  |
|  | AT1G15380 | 262603_at |  | 0004462 // lactoylglutathione lyase activity // inferred from sequence or structural similarity |
|  | AT1G74770 | 262218_at |  | 0005515 // protein binding // inferred from electronic annotation /// 0008270 // zinc ion binding // inferred from electronic annotation /// 0046872 // metal ion binding // inferred from electronic annotation |
|  | AT1G78000 | 262133_at | SULTR1;2 | 0005215 // transporter activity // inferred from electronic annotation /// 0008271 // secondary active sulfate transmembrane transporter activity // inferred from electronic annotation /// 0015116 // sulfate transmembrane transporter activity // inferred from sequence or structural similarity /// 0015293 // symporter activity // inferred from electronic annotation |
|  | AT1G80240 /// AT2G44100 | 262045_at |  | 0005093 // Rab GDP-dissociation inhibitor activity // inferred from electronic annotation /// 0005093 // Rab GDP-dissociation inhibitor activity // inferred from genetic interaction |
|  | AT1G29280 | 260882_at | WRKY65 | 0003677 // DNA binding // inferred from electronic annotation /// 0003700 // transcription factor activity // inferred from sequence or structural similarity /// 0003700 // transcription factor activity // inferred from electronic annotation /// 0043565 // sequence-specific DNA binding // inferred from electronic annotation |
|  | AT1G78340 | 260803_at | ATGSTU22 | 0004364 // glutathione transferase activity // inferred from sequence or structural similarity /// 0016740 // transferase activity // inferred from electronic annotation |
|  | AT1G72200 | 259854_at |  | 0005515 // protein binding // inferred from sequence or structural similarity /// 0005515 // protein binding // inferred from electronic annotation /// 0008270 // zinc ion binding // inferred from sequence or structural similarity /// 0008270 // zinc ion binding // inferred from electronic annotation /// 0046872 // metal ion binding // inferred from electronic annotation |
|  | AT3G11550 | 259291_at |  |  |
|  | AT3G05890 | 258751_at | RCI2B |  |
|  | AT3G21710 | 257946_at |  |  |
|  | AT3G23190 | 257924_at |  |  |
|  | AT3G29250 | 257774_at |  | 0003824 // catalytic activity // inferred from electronic annotation /// 0005488 // binding // inferred from electronic annotation /// 0005507 // copper ion binding // inferred from direct assay /// 0016491 // oxidoreductase activity // inferred from sequence or structural similarity /// 0016491 // oxidoreductase activity // inferred from electronic annotation |
|  | AT3G24240 | 257244_at |  | 0000166 // nucleotide binding // inferred from electronic annotation /// 0004672 // protein kinase activity // inferred from electronic annotation /// 0004674 // protein serine/threonine kinase activity // inferred from sequence or structural similarity /// 0004674 // protein serine/threonine kinase activity // inferred from electronic annotation /// 0004872 // receptor activity // inferred from electronic annotation /// 0005515 // protein binding // inferred from electronic annotation /// 0005524 // ATP binding // inferred from sequence or structural similarity /// 0005524 // ATP binding // inferred from electronic annotation /// 0016301 // kinase activity // inferred from electronic annotation /// 0016740 // transferase activity // inferred from electronic annotation |
|  | AT3G23800 | 257197_at |  | 0008430 // selenium binding // inferred from sequence or structural similarity /// 0008430 // selenium binding // inferred from electronic annotation |
|  | AT3G25820 /// AT3G25830 | 256994_s_at | ATTPS-CIN | 0000287 // magnesium ion binding // inferred from electronic annotation /// 0016829 // lyase activity // inferred from electronic annotation /// 0030145 // manganese ion binding // inferred from electronic annotation /// 0034768 // (E)-beta-ocimene synthase activity // inferred from direct assay /// 0046872 // metal ion binding // inferred from electronic annotation /// 0050551 // myrcene synthase activity // inferred from direct assay |
|  | AT3G22570 | 256935_at |  | 0008289 // lipid binding // inferred from sequence or structural similarity |
|  | AT1G30370 | 256306_at |  | 0004806 // triacylglycerol lipase activity // inferred from sequence or structural similarity /// 0004806 // triacylglycerol lipase activity // inferred from electronic annotation |
|  | AT4G07820 | 255160_at |  |  |
|  | AT4G12510 /// AT4G12520 | 254820_s_at |  | 0008289 // lipid binding // inferred from sequence or structural similarity |
|  | AT4G13860 | 254738_at |  | 0000166 // nucleotide binding // inferred from electronic annotation /// 0003676 // nucleic acid binding // inferred from electronic annotation /// 0003723 // RNA binding // inferred from sequence or structural similarity |
|  | AT4G18510 | 254644_at | CLE2 | 0005102 // receptor binding // inferred from sequence or structural similarity /// 0005515 // protein binding // inferred from sequence or structural similarity |
|  | AT4G21850 | 254387_at |  | 0008113 // peptide-methionine-(S)-S-oxide reductase activity // inferred from electronic annotation |
|  | AT4G22610 | 254326_at |  | 0008289 // lipid binding // inferred from sequence or structural similarity |
|  | AT4G26320 | 253957_at | AGP13 |  |
|  | AT4G30670 | 253582_at |  |  |
|  | AT4G31910 | 253483_at |  | 0016740 // transferase activity // inferred from sequence or structural similarity /// 0016747 // transferase activity, transferring acyl groups other than amino-acyl groups // inferred from electronic annotation |
|  | AT3G44320 | 252677_at | NIT3 | 0000257 // nitrilase activity // inferred from direct assay /// 0000257 // nitrilase activity // inferred from electronic annotation /// 0016787 // hydrolase activity // inferred from electronic annotation /// 0016810 // hydrolase activity, acting on carbon-nitrogen (but not peptide) bonds // inferred from electronic annotation /// 0080061 // indole-3-acetonitrile nitrilase activity // inferred from direct assay |
|  | AT3G45710 | 252537_at |  | 0005215 // transporter activity // inferred from sequence or structural similarity |
|  | AT3G52450 | 252045_at |  | 0004842 // ubiquitin-protein ligase activity // inferred from genetic interaction /// 0004842 // ubiquitin-protein ligase activity // inferred from electronic annotation /// 0005488 // binding // inferred from electronic annotation /// 0016874 // ligase activity // inferred from electronic annotation |
|  | AT3G54040 | 251918_at |  |  |
|  | AT3G54770 | 251857_at |  | 0000166 // nucleotide binding // inferred from electronic annotation /// 0003676 // nucleic acid binding // inferred from electronic annotation /// 0003723 // RNA binding // inferred from sequence or structural similarity |
|  | AT5G08240 | 250575_at |  |  |
|  | AT5G10130 | 250469_at |  |  |
|  | AT5G10580 | 250438_at |  |  |
|  | AT5G15130 | 250153_at | WRKY72 | 0003677 // DNA binding // inferred from electronic annotation /// 0003700 // transcription factor activity // inferred from sequence or structural similarity /// 0003700 // transcription factor activity // inferred from electronic annotation /// 0043565 // sequence-specific DNA binding // inferred from electronic annotation |
|  | AT5G17820 | 250059_at |  | 0004601 // peroxidase activity // inferred from sequence or structural similarity /// 0004601 // peroxidase activity // inferred from electronic annotation /// 0005506 // iron ion binding // inferred from electronic annotation /// 0005509 // calcium ion binding // inferred from electronic annotation /// 0009055 // electron carrier activity // inferred from electronic annotation /// 0016491 // oxidoreductase activity // inferred from electronic annotation /// 0020037 // heme binding // inferred from electronic annotation /// 0046872 // metal ion binding // inferred from electronic annotation |
|  | AT5G24410 | 249729_at |  | 0016787 // hydrolase activity // inferred from electronic annotation /// 0017057 // 6-phosphogluconolactonase activity // inferred from electronic annotation |
|  | AT5G40510 | 249358_at |  |  |
|  | AT5G42180 | 249227_at |  | 0004601 // peroxidase activity // inferred from sequence or structural similarity /// 0004601 // peroxidase activity // inferred from electronic annotation /// 0005506 // iron ion binding // inferred from electronic annotation /// 0005509 // calcium ion binding // inferred from electronic annotation /// 0009055 // electron carrier activity // inferred from electronic annotation /// 0016491 // oxidoreductase activity // inferred from electronic annotation /// 0020037 // heme binding // inferred from electronic annotation /// 0046872 // metal ion binding // inferred from electronic annotation |
|  | AT5G43180 | 249136_at |  |  |
|  | AT5G43350 /// AT5G43370 | 249152_s_at | ATPT1 | 0005315 // inorganic phosphate transmembrane transporter activity // inferred from direct assay /// 0005315 // inorganic phosphate transmembrane transporter activity // inferred from sequence or structural similarity /// 0005315 // inorganic phosphate transmembrane transporter activity // inferred from electronic annotation /// 0005351 // sugar:hydrogen symporter activity // inferred from sequence or structural similarity /// 0015114 // phosphate transmembrane transporter activity // inferred from mutant phenotype /// 0015114 // phosphate transmembrane transporter activity // inferred from sequence or structural similarity /// 0015144 // carbohydrate transmembrane transporter activity // inferred from sequence or structural similarity /// 0015293 // symporter activity // inferred from electronic annotation |
|  | AT5G44380 | 249045_at |  | 0003824 // catalytic activity // inferred from electronic annotation /// 0009055 // electron carrier activity // inferred from sequence or structural similarity /// 0016491 // oxidoreductase activity // inferred from electronic annotation /// 0050660 // FAD binding // inferred from electronic annotation |
|  | AT5G44610 | 249009_at | MAP18 | 0008017 // microtubule binding // inferred from direct assay |
|  | AT5G44920 | 249033_at |  | 0004888 // transmembrane receptor activity // inferred from electronic annotation |
|  | AT5G45070 | 248978_at | ATPP2-A8 | 0004888 // transmembrane receptor activity // inferred from electronic annotation /// 0030246 // carbohydrate binding // inferred from sequence or structural similarity |
|  | AT5G47450 | 248790_at | AtTIP2;3 | 0005215 // transporter activity // inferred from electronic annotation /// 0015200 // methylammonium transmembrane transporter activity // inferred from direct assay /// 0015250 // water channel activity // inferred from sequence or structural similarity /// 0051739 // ammonia transporter activity // inferred from genetic interaction |
|  | AT5G47950 | 248723_at |  | 0016740 // transferase activity // inferred from sequence or structural similarity /// 0016747 // transferase activity, transferring acyl groups other than amino-acyl groups // inferred from electronic annotation |
|  | AT5G48430 | 248703_at |  | 0004190 // aspartic-type endopeptidase activity // inferred from electronic annotation |
|  | AT5G53250 | 248252_at | AGP22 |  |
|  | AT5G60660 | 247586_at | PIP2;4 | 0005215 // transporter activity // inferred from electronic annotation /// 0015250 // water channel activity // inferred from sequence or structural similarity |
|  | AT5G62340 | 247477_at |  | 0004857 // enzyme inhibitor activity // inferred from electronic annotation /// 0030599 // pectinesterase activity // inferred from electronic annotation /// 0046910 // pectinesterase inhibitor activity // inferred from sequence or structural similarity |
|  | AT5G63600 | 247333_at |  | 0005506 // iron ion binding // inferred from electronic annotation /// 0016491 // oxidoreductase activity // inferred from electronic annotation /// 0045431 // flavonol synthase activity // inferred from sequence or structural similarity |
|  | AT5G64100 | 247297_at |  | 0004601 // peroxidase activity // inferred from sequence or structural similarity /// 0004601 // peroxidase activity // inferred from electronic annotation /// 0005506 // iron ion binding // inferred from electronic annotation /// 0005509 // calcium ion binding // inferred from electronic annotation /// 0009055 // electron carrier activity // inferred from electronic annotation /// 0016491 // oxidoreductase activity // inferred from electronic annotation /// 0020037 // heme binding // inferred from electronic annotation /// 0046872 // metal ion binding // inferred from electronic annotation |
|  | AT5G64905 | 247215_at | PROPEP3 |  |
|  | AT5G66690 | 247059_at | UGT72E2 | 0008194 // UDP-glycosyltransferase activity // inferred from sequence or structural similarity /// 0016740 // transferase activity // inferred from electronic annotation /// 0016757 // transferase activity, transferring glycosyl groups // inferred from sequence or structural similarity /// 0016757 // transferase activity, transferring glycosyl groups // inferred from electronic annotation /// 0016758 // transferase activity, transferring hexosyl groups // inferred from electronic annotation /// 0047209 // coniferyl-alcohol glucosyltransferase activity // inferred from direct assay /// 0047209 // coniferyl-alcohol glucosyltransferase activity // inferred from mutant phenotype |
|  | AT1G77330 | 246390_at |  | 0005506 // iron ion binding // inferred from electronic annotation /// 0016491 // oxidoreductase activity // inferred from electronic annotation |
|  | AT4G15390 | 245555_at |  | 0016740 // transferase activity // inferred from sequence or structural similarity /// 0016747 // transferase activity, transferring acyl groups other than amino-acyl groups // inferred from electronic annotation |
|  | AT2G45430 | 245139_at |  | 0003677 // DNA binding // inferred from electronic annotation |
