# Supplementary material for: Identification, Validation and Utilization of Novel Nematode-Responsive Root-Specific Promoters in Arabidopsis for Inducing Host-Delivered RNAi Mediated Root-Knot Nematode Resistance
Source: Front Plant Sci. 2017 Dec 12;8:2049. doi: 10.3389/fpls.2017.02049 (PMC5733009; doi:10.3389/fpls.2017.02049)
Supplement: Supplementary Table 3 — Genes that were found to be up-regulated, in Arabidopsis thaliana on Meloidogyne incognita infection at the early and late stages, through meta-analysis. [file Table3.DOCX]

**S3 Table. Genes that were found to be up-regulated, in *Arabidopsis thaliana* on *Meloidogyne incognita* infection at the early and late stages, through meta-analysis.**

|  | **AGI** | **Probe** | **Annotation** |
| --- | --- | --- | --- |
|  | AT1G03820 | 265083_at | similar to E6-4 [Gossypium hirsutum] (GB:AAY43793.1); similar to E6-3 [Gossypium hirsutum] (GB:AAY43792.1); similar to E6 (GB:AAB03081.1) |
|  | AT1G70710 | 262654_at | Endo-1, 4- beta-glucanase (involved in cell elongation) |
|  | AT1G14840 | 262847_at | ATMAP70-4 (microtubule-associated proteins 70-4); microtubule binding |
|  | AT1G18250 | 256125_at | ATLP-1 (Arabidopsis thaumatin-like protein 1) |
|  | AT1G18670 | 261427_at | IBS1 (IMPAIRED IN BABA-INDUCED STERILITY 1); kinase |
|  | AT1G31320 | 257467_at | LBD4 (LOB DOMAIN-CONTAINING PROTEIN 4) |
|  | AT1G31710 | 246602_at | copper amine oxidase, putative |
|  | AT1G43300;  AT3G43460 | 264414_s_at | [AT1G43300, transposable element gene];[AT3G43460, transposable element gene] |
|  | AT1G60630 | 264939_at | leucine-rich repeat family protein |
|  | AT1G63710 | 260241_at | CYP86A7 (cytochrome P450, family 86, subfamily A, polypeptide 7); oxygen binding |
|  | AT1G64450 | 262002_at | proline-rich family protein |
|  | AT2G03090 | 266770_at | ATEXPA15 (ARABIDOPSIS THALIANA EXPANSIN A15) |
|  | AT2G28950 | 266790_at | ATEXPA6 (ARABIDOPSIS THALIANA EXPANSIN A6) |
|  | AT2G32860 | 267645_at | glycosyl hydrolase family 1 protein |
|  | AT2G36570 | 263913_at | leucine-rich repeat transmembrane protein kinase, putative |
|  | AT2G39700 | 267590_at | ATEXPA4 (ARABIDOPSIS THALIANA EXPANSIN A4) |
|  | AT2G46110 | 266598_at | KPHMT1/PANB1 (KETOPANTOATE HYDROXYMETHYLTRANSFERASE 1); 3-methyl-2-oxobutanoate hydroxymethyltransferase |
|  | AT3G02885 | 258618_at | GASA5 (GAST1 PROTEIN HOMOLOG 5) |
|  | AT3G07540 | 259021_at | formin homology 2 domain-containing protein / FH2 domain-containing protein |
|  | AT3G15170 | 256857_at | CUC1 (CUP-SHAPED COTYLEDON1); transcription factor |
|  | AT3G16130 | 258330_at | ATROPGEF13/ROPGEF13 (KINASE PARTNER PROTEIN-LIKE); Rho guanyl-nucleotide exchange factor/ |
|  | AT3G17680 | 258376_at | similar to unknown protein [Arabidopsis thaliana] (TAIR:AT1G48405.1); similar to hypothetical protein [Vitis vinifera] (GB:CAN67913.1); contains InterPro domain KIP1-like (InterPro:IPR011684) |
|  | AT3G18280 | 257066_at | protease inhibitor/seed storage/lipid transfer protein (LTP) family protein |
|  | AT3G22120 | 256825_at | CWLP (CELL WALL-PLASMA MEMBRANE LINKER PROTEIN); lipid binding |
|  | AT3G61820 | 251287_at | aspartyl protease family protein |
|  | AT3G63440 | 251178_at | ATCKX6/ATCKX7/CKX6 (CYTOKININ OXIDASE/DEHYDROGENASE 6); cytokinin dehydrogenase |
|  | AT4G09010 | 255078_at | APX4 (ASCORBATE PEROXIDASE 4); peroxidase |
|  | AT4G18910 | 254608_at | NIP1;2/NLM2 (NOD26-like intrinsic protein 1;2); water channel |
|  | AT4G24780 | 254119_at | pectate lyase family protein |
|  | AT4G28250 | 253815_at | ATEXPB3 (ARABIDOPSIS THALIANA EXPANSIN B3) |
|  | AT4G34160 | 253270_at | CYCD3/CYCD3;1/D3 (CYCLIN D3;1); cyclin-dependent protein kinase regulator/ protein binding |
|  | AT4G37450 | 253050_at | AGP18 (Arabinogalactan protein 18) |
|  | AT4G38770 | 252971_at | PRP4 (PROLINE-RICH PROTEIN 4) |
|  | AT4G39590 | 252910_at | kelch repeat-containing F-box family protein |
|  | AT5G05940 | 250756_at | ATROPGEF5/ROPGEF5 (KINASE PARTNER PROTEIN-LIKE); Rho guanyl-nucleotide exchange factor/ |
|  | AT5G11510 | 250386_at | MYB3R-4 (C-MYB-LIKE TRANSCRIPTION FACTOR 3R-4, myb domain protein 3R-4); DNA binding / transcription coactivator/ transcription factor |
|  | AT5G12270 | 245204_at | oxidoreductase, 2OG-Fe(II) oxygenase family protein |
|  | AT5G44400 | 249046_at | FAD-binding domain-containing protein |
|  | AT5G45400 | 248940_at | replication protein, putative |
|  | AT5G49630 | 248619_at | AAP6 (AMINO ACID PERMEASE 6); amino acid transmembrane transporter |
|  | AT2G25930 | 248513_at | Early Flowering 3, ELF3, PYK20 |
|  | AT5G51550 | 248419_at | phosphate-responsive 1 family protein |
