# Supplementary material for: Identification, Validation and Utilization of Novel Nematode-Responsive Root-Specific Promoters in Arabidopsis for Inducing Host-Delivered RNAi Mediated Root-Knot Nematode Resistance
Source: Front Plant Sci. 2017 Dec 12;8:2049. doi: 10.3389/fpls.2017.02049 (PMC5733009; doi:10.3389/fpls.2017.02049)
Supplement: Supplementary Table 4 — NRRS genes identified using standalone analysis. [file Table4.DOCX]

**S4 Table. NRRS genes identified using standalone analysis.**

| **S.NO** | **AGI** | **Gene Model Type** | **Primary Gene Annotation or function** |
| --- | --- | --- | --- |
|  | AT5G38020 | protein_coding | salicylic acid carboxyl methyltransferase (SAMT), benzoic acid carboxyl methyltransferase (BAMT) |
|  | AT1G80320 | protein_coding | oxoglutarate (2OG) and Fe(II)-dependent oxygenase superfamily protein (not validate) |
|  | AT2G29660 | protein_coding | zinc finger (C2H2 type) family protein |
|  | AT4G01390 | protein_coding | TRAF-like family protein; |
|  | AT2G16005 | protein_coding | INTERACTOR OF SYNAPTOTAGMIN1, ROSY1 |
|  | AT2G40230 | protein_coding | HXXXD-type acyl-transferase family protein |
|  | AT3G25290 | protein_coding | Auxin-responsive family protein |
|  | AT1G68360 | protein_coding | GLABROUS INFLORESCENCE STEMS 3 (GIS3) |
|  | AT2G39510 | protein_coding | USUALLY MULTIPLE ACIDS MOVE IN AND OUT TRANSPORTERS 14 (UMAMIT14) |
|  | AT1G52820 | protein_coding | 2-oxoglutarate (2OG) and Fe(II)-dependent oxygenase superfamily protein |
|  | AT4G29270 | protein_coding | HAD superfamily, subfamily IIIB acid phosphatase |
|  | AT2G01610 | protein_coding | Plant invertase/pectin methylesterase inhibitor superfamily protein |
|  | AT5G47980 | protein_coding | HXXXD-type acyl-transferase family protein;(source:Araport11) |
|  | AT4G36430 | protein_coding | Peroxidase superfamily protein |
|  | AT1G74770 | protein_coding | zinc ion binding protein |
|  | AT2G42250 | protein_coding | CYTOCHROME P450, FAMILY 712, SUBFAMILY A, POLYPEPTIDE 1 (CYP712A1) |
|  | AT1G67110 | protein_coding | CYTOCHROME P450, FAMILY 735, SUBFAMILY A, POLYPEPTIDE 2 (CYP735A2) |
|  | AT5G58860 | protein_coding | CYTOCHROME P450, FAMILY 86, SUBFAMILY A, POLYPEPTIDE 1 (CYP86A1) |
|  | AT4G30140 | protein_coding | CUTICLE DESTRUCTING FACTOR 1 (CDEF1) |
|  | AT5G56320 | protein_coding | EXPANSIN A14 (EXPA14) |
|  | AT5G56080 | protein_coding | NICOTIANAMINE SYNTHASE 2 (NAS2) |
|  | AT1G30510 | protein_coding | ROOT FNR 2 (RFNR2), Encodes a root-type ferredoxin:NADP(H) oxidoreductase |
|  | AT1G78120 | protein_coding | TETRATRICOPEPTIDE REPEAT 12 (TPR12) |
|  | AT3G54260 | protein_coding | TRICHOME BIREFRINGENCE-LIKE 36 (TBL36) |
|  | AT5G50200 | protein_coding | WOUND-RESPONSIVE 3 (WR3) |
