# Supplementary material for: Identification, Validation and Utilization of Novel Nematode-Responsive Root-Specific Promoters in Arabidopsis for Inducing Host-Delivered RNAi Mediated Root-Knot Nematode Resistance
Source: Front Plant Sci. 2017 Dec 12;8:2049. doi: 10.3389/fpls.2017.02049 (PMC5733009; doi:10.3389/fpls.2017.02049)
Supplement: Supplementary Table 5 — NRRS genes identified using meta-analysis. [file Table5.DOCX]

**S5 Table. NRRS genes identified using meta-analysis.**

| **S. No.** | **AGI** | **Gene Model Type** | **Primary Gene Symbol** |
| --- | --- | --- | --- |
|  | AT2G25150 | protein_coding | HXXXD-type acyl-transferase family protein |
|  | AT1G72660 | protein_coding | P-loop containing nucleoside triphosphate hydrolases superfamily protein |
|  | AT1G26530 | protein_coding | PIN domain-like family protein |
|  | AT2G22930 | protein_coding | UDP-Glycosyltransferase superfamily protein |
|  | AT5G37990 | protein_coding | S-adenosyl-L-methionine-dependent methyltransferase superfamily protein |
|  | AT2G18140 | protein_coding | Peroxidase superfamily protein |
|  | AT5G47980 | protein_coding | HXXXD-type acyl-transferase family protein |
|  | AT2G18150 | protein_coding | Peroxidase superfamily protein |
|  | AT1G74770 | protein_coding | zinc ion binding protein |
|  | AT5G38020 | protein_coding | S-adenosyl-L-methionine-dependent methyltransferase superfamily protein |
|  | AT2G16005 | protein_coding | \| \| INTERACTOR OF SYNAPTOTAGMIN1, ROSY1 \| \| --- \| \| \| --- \| --- \| |
|  | AT5G58780 | protein_coding | TCPT5, ATHEPS, CIS -PRENYLTRANSFERASE 5, CPT5, HEPS, HEPTAPRENYL DIPHOSPHATE SYNTHASE |
|  | AT1G48670 | protein_coding | auxin-responsive GH3 family protein |
|  | AT4G36430 | protein_coding | Peroxidase superfamily protein |
|  | AT4G11190 | protein_coding | Disease resistance-responsive (dirigent-like protein) family protein |
|  | AT3G29775 | transposable_element_gene | Transposable element gene |
|  | AT4G21340 | protein_coding | (B70), basic helix-loop-helix (bHLH) DNA-binding superfamily protein |
|  | AT5G36140 | protein_coding | CYTOCHROME P450, FAMILY 716, SUBFAMILY A, POLYPEPTIDE 2 (CYP716A2) |
|  | AT1G01480 | protein_coding | 1-AMINO-CYCLOPROPANE-1-CARBOXYLATE SYNTHASE 2 (ACS2) |
|  | AT5G56320 | protein_coding | EXPANSIN A14 (EXPA14) |
|  | AT3G06020 | protein_coding | FANTASTIC FOUR 4 (FAF4) |
|  | AT4G32890 | protein_coding | GATA TRANSCRIPTION FACTOR 9 (GATA9) |
|  | AT4G11050 | protein_coding | GLYCOSYL HYDROLASE 9C3 (GH9C3) |
|  | AT5G59720 | protein_coding | HEAT SHOCK PROTEIN 18.2 (HSP18.2) |
|  | AT1G80100 | protein_coding | HISTIDINE PHOSPHOTRANSFER PROTEIN 6 (HP6) |
|  | AT2G30210 | protein_coding | LACCASE 3 (LAC3) |
|  | AT5G56080 | protein_coding | NICOTIANAMINE SYNTHASE 2 (NAS2) |
|  | AT1G30510 | protein_coding | ROOT FNR 2 (RFNR2) |
|  | AT5G40390 | protein_coding | SEED IMBIBITION 1-LIKE (SIP1) |
|  | AT5G60200 | protein_coding | TARGET OF MONOPTEROS 6 (TMO6) |
|  | AT5G48010 | protein_coding | THALIANOL SYNTHASE 1 (THAS1) |
|  | AT3G51030 | protein_coding | THIOREDOXIN H-TYPE 1 (TRX1) |
|  | AT3G44990 | protein_coding | XYLOGLUCAN ENDO-TRANSGLYCOSYLASE-RELATED 8 (XTR8) |
|  | AT5G48070 | protein_coding | XYLOGLUCAN ENDOTRANSGLUCOSYLASE/HYDROLASE 20 (XTH20) |
