# Supplementary material for: Identification, Validation and Utilization of Novel Nematode-Responsive Root-Specific Promoters in Arabidopsis for Inducing Host-Delivered RNAi Mediated Root-Knot Nematode Resistance
Source: Front Plant Sci. 2017 Dec 12;8:2049. doi: 10.3389/fpls.2017.02049 (PMC5733009; doi:10.3389/fpls.2017.02049)
Supplement: Supplementary Table 6 — NRRS genes (8 nos.) common between the standalone and meta-analysis. [file Table6.DOCX]

**S6 Table. NRRS genes (8 nos.) common between the standalone and meta-analysis.**

| **S. No.** | **AGI** | **Gene Model Type** | **Primary Gene Symbol** |
| --- | --- | --- | --- |
|  | AT5G38020 | protein_coding |  |
|  | AT2G16005 | protein_coding |  |
|  | AT5G47980 | protein_coding |  |
|  | AT4G36430 | protein_coding |  |
|  | AT1G74770 | protein_coding |  |
|  | AT5G56320 | protein_coding | EXPANSIN A14 (EXPA14) |
|  | AT5G56080 | protein_coding | NICOTIANAMINE SYNTHASE 2 (NAS2) |
|  | AT1G30510 | protein_coding | ROOT FNR 2 (RFNR2) |
