# Supplementary material for: Identification, Validation and Utilization of Novel Nematode-Responsive Root-Specific Promoters in Arabidopsis for Inducing Host-Delivered RNAi Mediated Root-Knot Nematode Resistance
Source: Front Plant Sci. 2017 Dec 12;8:2049. doi: 10.3389/fpls.2017.02049 (PMC5733009; doi:10.3389/fpls.2017.02049)

## Slide 1
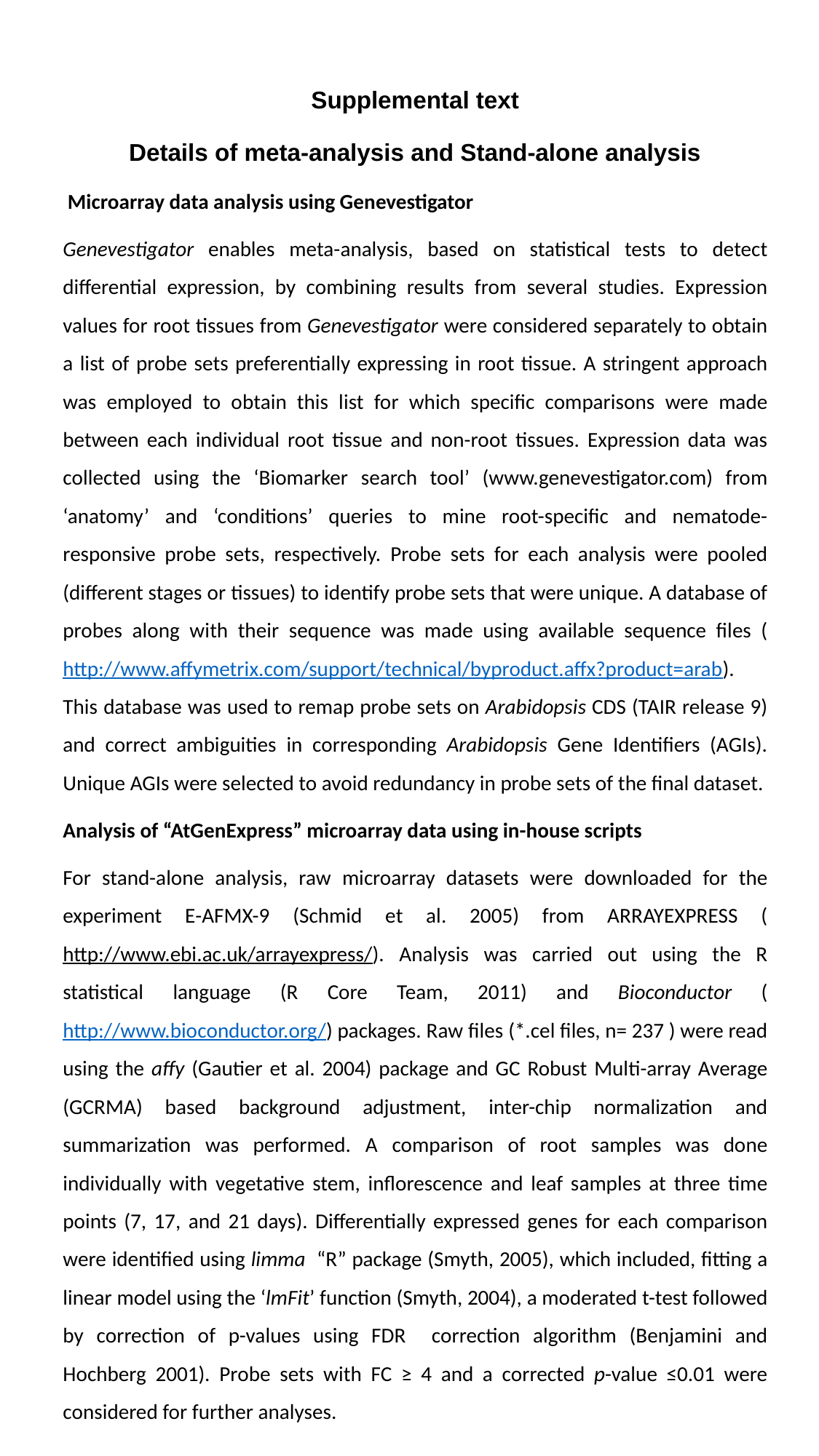

Supplemental text
Details of meta-analysis and Stand-alone analysis
 Microarray data analysis using Genevestigator
Genevestigator enables meta-analysis, based on statistical tests to detect differential expression, by combining results from several studies. Expression values for root tissues from Genevestigator were considered separately to obtain a list of probe sets preferentially expressing in root tissue. A stringent approach was employed to obtain this list for which specific comparisons were made between each individual root tissue and non-root tissues. Expression data was collected using the ‘Biomarker search tool’ (www.genevestigator.com) from ‘anatomy’ and ‘conditions’ queries to mine root-specific and nematode-responsive probe sets, respectively. Probe sets for each analysis were pooled (different stages or tissues) to identify probe sets that were unique. A database of probes along with their sequence was made using available sequence files (http://www.affymetrix.com/support/technical/byproduct.affx?product=arab). This database was used to remap probe sets on Arabidopsis CDS (TAIR release 9) and correct ambiguities in corresponding Arabidopsis Gene Identifiers (AGIs). Unique AGIs were selected to avoid redundancy in probe sets of the final dataset.
Analysis of “AtGenExpress” microarray data using in-house scripts
For stand-alone analysis, raw microarray datasets were downloaded for the experiment E-AFMX-9 (Schmid et al. 2005) from ARRAYEXPRESS (http://www.ebi.ac.uk/arrayexpress/). Analysis was carried out using the R statistical language (R Core Team, 2011) and Bioconductor (http://www.bioconductor.org/) packages. Raw files (*.cel files, n= 237 ) were read using the affy (Gautier et al. 2004) package and GC Robust Multi-array Average (GCRMA) based background adjustment, inter-chip normalization and summarization was performed. A comparison of root samples was done individually with vegetative stem, inflorescence and leaf samples at three time points (7, 17, and 21 days). Differentially expressed genes for each comparison were identified using limma “R” package (Smyth, 2005), which included, fitting a linear model using the ‘lmFit’ function (Smyth, 2004), a moderated t-test followed by correction of p-values using FDR correction algorithm (Benjamini and Hochberg 2001). Probe sets with FC ≥ 4 and a corrected p-value ≤0.01 were considered for further analyses.

## Slide 2
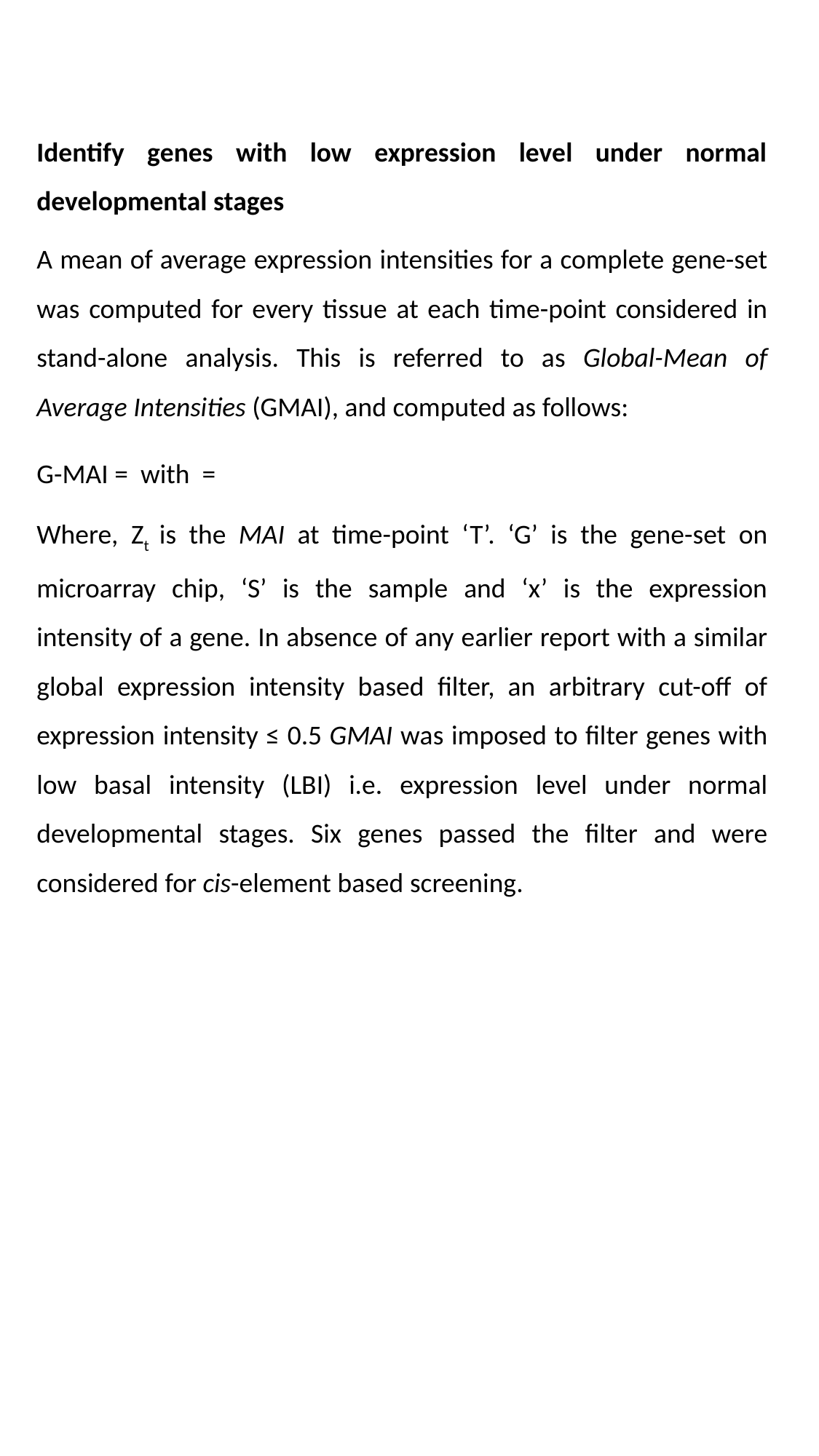

Supplement: Supplementary Text Presentation 1 — Details of meta-analysis and stand-alone analysis. [file Presentation1.PPTX]
