# Supplementary material for: Identification, Validation and Utilization of Novel Nematode-Responsive Root-Specific Promoters in Arabidopsis for Inducing Host-Delivered RNAi Mediated Root-Knot Nematode Resistance
Source: Front Plant Sci. 2017 Dec 12;8:2049. doi: 10.3389/fpls.2017.02049 (PMC5733009; doi:10.3389/fpls.2017.02049)
Supplement: Supplementary Figure Presentation 1 — MEME analysis using promoter region of 51 nematode-responsive root-specific genes. [file Presentation2.PPTX]

## Slide 1
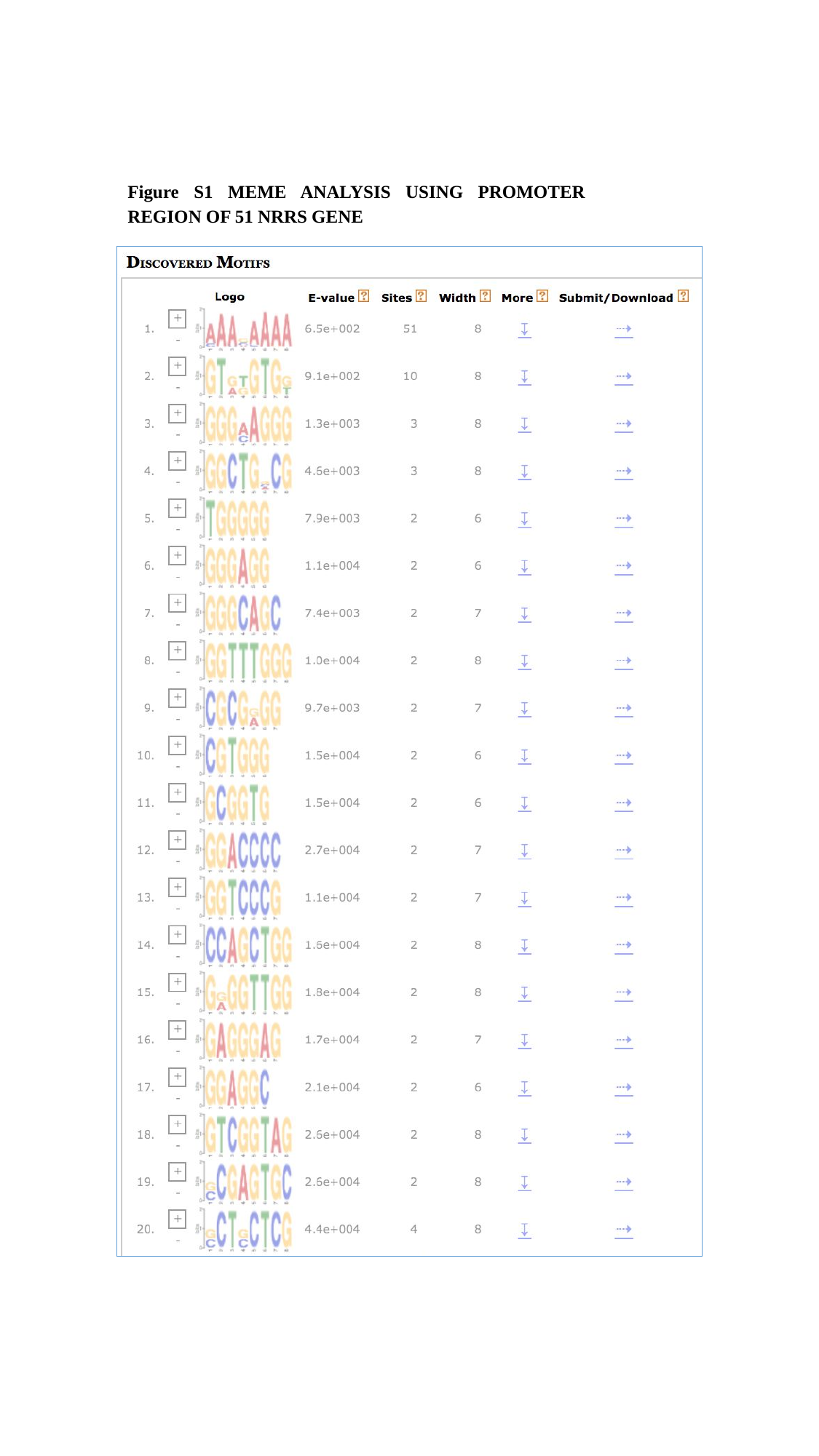

Figure S1 MEME ANALYSIS USING PROMOTER REGION OF 51 NRRS GENE

## Slide 2
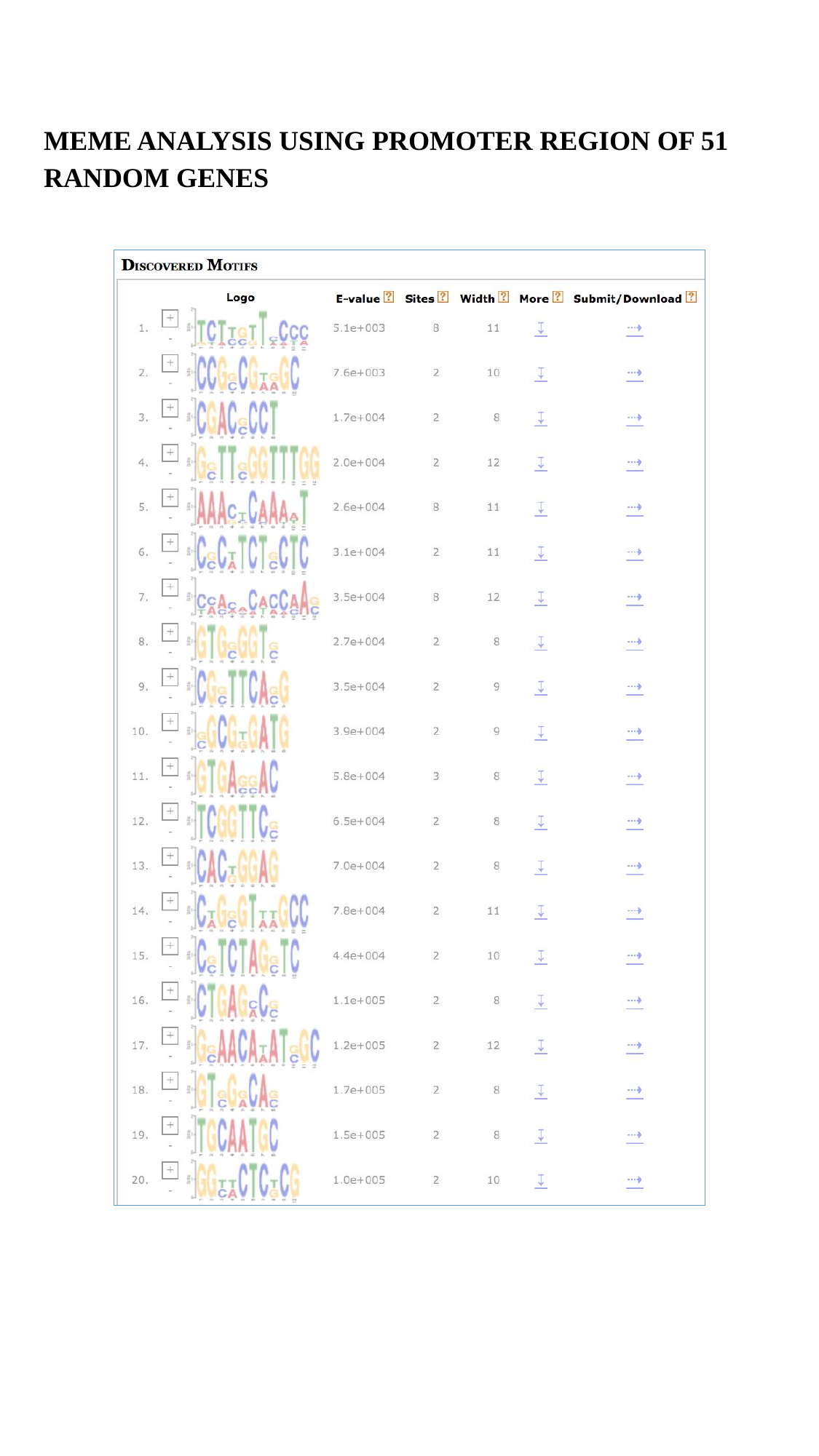

MEME ANALYSIS USING PROMOTER REGION OF 51 RANDOM GENES

## Slide 3
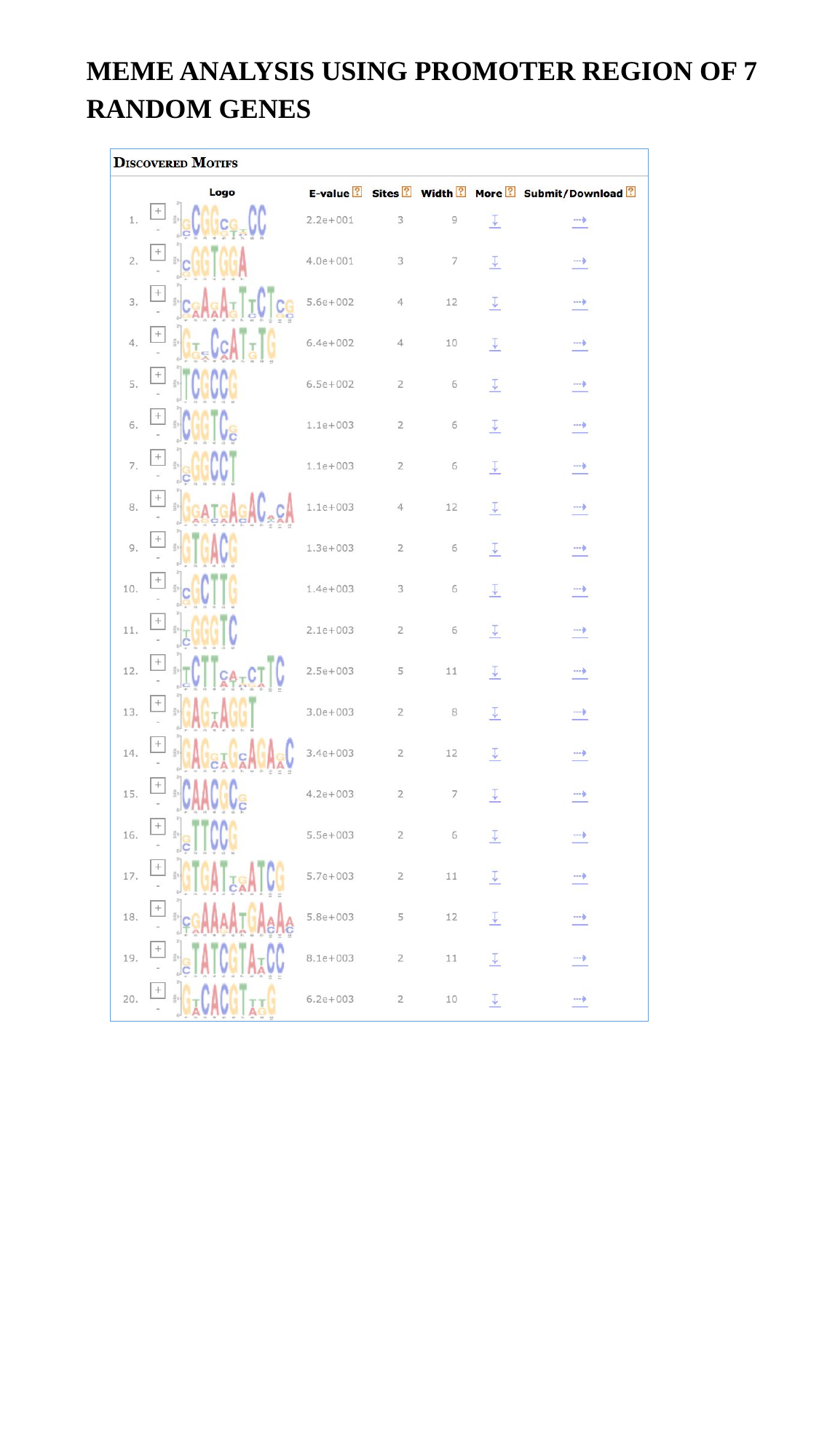

MEME ANALYSIS USING PROMOTER REGION OF 7 RANDOM GENES
