# Supplementary figures and images for: Identification, Validation and Utilization of Novel Nematode-Responsive Root-Specific Promoters in Arabidopsis for Inducing Host-Delivered RNAi Mediated Root-Knot Nematode Resistance
Source: Front Plant Sci. 2017 Dec 12;8:2049. doi: 10.3389/fpls.2017.02049 (PMC5733009; doi:10.3389/fpls.2017.02049)

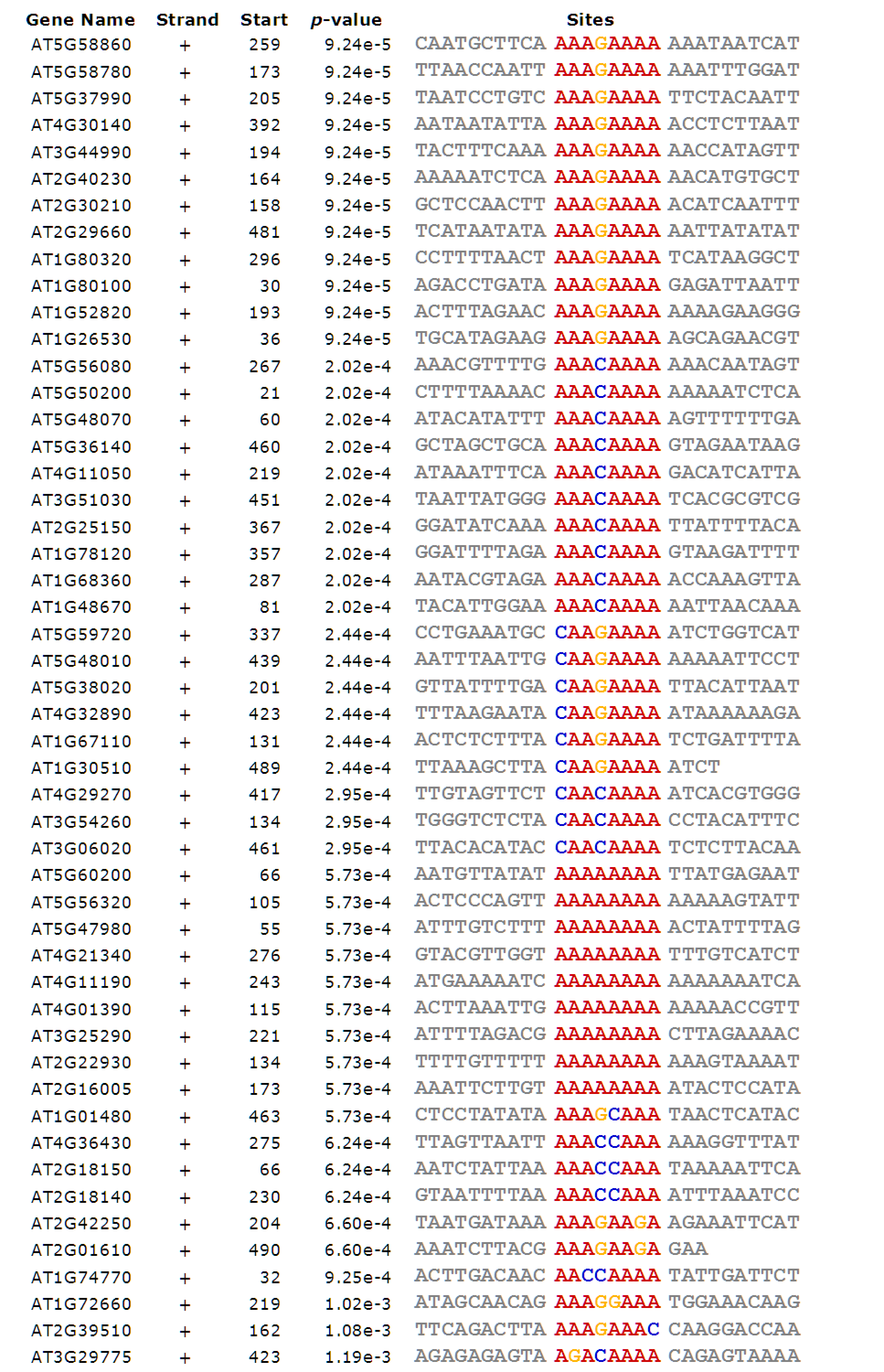

Supplement: Supplementary Figure 1 — MEME output for 51 NRRS genes for motif AAAxAAAA. [file Image1.PNG]
